# Combination of plant metabolites hinders starch digestion and glucose absorption while facilitating insulin sensitivity to diabetes

**DOI:** 10.3389/fphar.2024.1362150

**Published:** 2024-06-05

**Authors:** Xin Huang, Kaihuang Lin, Sinian Liu, Junxiong Yang, Haowei Zhao, Xiao-Hui Zheng, May-Jywan Tsai, Chun-Sheng Chang, Liyue Huang, Ching-Feng Weng

**Affiliations:** ^1^ Functional Physiology Section, Department of Basic Medical Science, Xiamen Medical College, Xiamen, China; ^2^ Institute of Respiratory Disease, Department of Basic Medical Science, Xiamen Medical College, Xiamen, China; ^3^ Department of Neurosurgery, Neurological Institute, Taipei Veterans General Hospital, Taipei, Taiwan; ^4^ Department of Biotechnology and Food Technology, Southern Taiwan University of Science and Technology, Tainan, Taiwan

**Keywords:** antihyperglycemia, combinatory chemistry, phytopharmaceutical, polyphenol, *in silico*, multitarget

## Abstract

**Introduction:**

Diabetes mellitus (DM) is a common endocrine disease resulting from interactions between genetic and environmental factors. Type II DM (T2DM) accounts for approximately 90% of all DM cases. Current medicines used in the treatment of DM have some adverse or undesirable effects on patients, necessitating the use of alternative medications.

**Methods:**

To overcome the low bioavailability of plant metabolites, all entities were first screened through pharmacokinetic, network pharmacology, and molecular docking predictions. Experiments were further conducted on a combination of antidiabetic phytoactive molecules (rosmarinic acid, RA; luteolin, Lut; resveratrol, RS), along with *in vitro* evaluation (α-amylase inhibition assay) and diabetic mice tests (oral glucose tolerance test, OGTT; oral starch tolerance test, OSTT) for maximal responses to validate starch digestion and glucose absorption while facilitating insulin sensitivity.

**Results:**

The results revealed that the combination of metabolites achieved all required criteria, including ADMET, drug likeness, and Lipinski rule. To determine the mechanisms underlying diabetic hyperglycemia and T2DM treatments, network pharmacology was used for regulatory network, PPI network, GO, and KEGG enrichment analyses. Furthermore, the combined metabolites showed adequate *in silico* predictions (α-amylase, α-glucosidase, and pancreatic lipase for improving starch digestion; SGLT-2, AMPK, glucokinase, aldose reductase, acetylcholinesterase, and acetylcholine M2 receptor for mediating glucose absorption; GLP-1R, DPP-IV, and PPAR-γ for regulating insulin sensitivity), *in vitro* α-amylase inhibition, and *in vivo* efficacy (OSTT versus acarbose; OGTT versus metformin and insulin) as nutraceuticals against T2DM.

**Discussion:**

The results demonstrate that the combination of RA, Lut, and RS could be exploited for multitarget therapy as prospective antihyperglycemic phytopharmaceuticals that hinder starch digestion and glucose absorption while facilitating insulin sensitivity.

## 1 Introduction

Diabetes mellitus (DM) is a widespread metabolic disease with a rapidly growing global population; it is characterized by chronic hyperglycemia resulting from inadequate insulin secretion and/or insulin action (Ozougwu et al., 2013; Thompson and Kanamarlapudi, 2013) and is attributable to the interactions between genetic and environmental factors. Type II DM (T2DM) is also designated as non-insulin-dependent DM (NIDDM) and is often known to occur as a consequence of excess blood glucose (hyperglycemia) caused by dysfunctional β-cells and insulin resistance. T2DM is the major form of diabetes, as an estimated 90% of DM patients are diagnosed with this form. The global incidence of DM continues to increase, and it is expected that there will be more than 590 million patients with this disorder by 2035 (Ozougwu et al., 2013; Guariguata et al., 2014). It is known that DM can cause several other complications, such as cardiovascular diseases, ischemic heart disease, obesity, peripheral vascular disease, stroke, retinopathy, neuropathy, nephropathy, diabetic foot ulcers, and a variety of heterogeneous diseases, based on abnormalities in the relative metabolic pathways (Boulton et al., 2005; Punthakee et al., 2018; Swilam et al., 2022), with T2DM causing over 95% of these comorbidities (Punthakee et al., 2018). Although various types of oral hyperglycemic drugs (e.g., acarbose, miglitol, voglibose, metformin**,** and sulfonylureas) are available for the treatment of diabetes (Spínola et al., 2019), these approved synthetic drugs cannot be applied for long-term glycemic control or reversal of comorbidity progression; their side effects or adverse reactions are also usually downplayed, coupled with the cost, which makes their access especially challenging for the low-income population in developing and underdeveloped countries. Therefore, there is an urgency for developing alternative therapeutics with limited associated shortcomings (Takayanagi et al., 2011). This is a major health issue in today’s society and is significantly linked with the socioeconomic difficulties experienced worldwide.

The documentation of natural products that moderate blood glucose levels can possibly accelerate exploitation of mild interventions like folk or herbal medicines as well as functional foods in the treatment of chronic diseases, including diabetes. In comparison, active metabolites from synthetic sources, extracts, or natural products from natural sources such as botanical drugs and plant metabolites that are commonly safe, easy to locate, easily accessible, and reasonably inexpensive, with low incidence of adverse effects must be prioritized to facilitate the surging diversion into phytomedicine (Wais et al., 2012; Bizzarri et al., 2020). Polyphenols are a large class of metabolites deemed to have multiple biological properties, such as antioxidant, cytotoxic, anti-inflammatory, antihypertensive, and antidiabetic functions (Rana et al., 2022). For instance, curcumin has been widely shown to be a popular antidiabetic, but its limitations like poor absorption, rapid metabolism and elimination, and low concentrations in the plasma and target tissue are considered to be obstacles in treatment (Pivari et al., 2019). It has been noted that monotarget therapy with natural polyphenols failed to manage blood glucose levels and other comorbidities; therefore, the combined use (polytherapy) of polyphenols has become a common practice (Boonrueng et al., 2022).

The flavone luteolin (3′, 4′, 5, 7-tetrahydroxyflavone, Lut) is a naturally occurring secondary metabolite present in various plants, such as celery, chrysanthemum flowers, sweet bell peppers, carrots, onion leaves, broccoli, and parsley. It has anti-inflammatory and antioxidant activities, and it increases glucose metabolism by potentiating insulin sensitivity while enhancing β-cell function and mass during a hyperglycemic clamp (Daily et al., 2021). However, one study revealed that Lut has limited bioavailability that consequently affects its biological properties and efficacy (Taheri et al., 2021). Resveratrol (3,5,4′-trihydroxy-trans-stilbene, RS) is a naturally occurring polyphenolic stilbene compound found in more than 70 plant species and their products, such as grapes, peanuts, mulberries, bilberries, blueberries, cranberries, and spruce, as well as other plant roots, leaves, and fruits in response to biotic and abiotic factors. It has been shown to have numerous biological activities, such as antitumor, antioxidant, antiviral, and phytoestrogenic properties**.** The effective use of RS is restricted by its poor solubility, photosensitivity, and rapid metabolism, which strongly undermine its bioavailability and bioactivity (Santos et al., 2019). Rosmarinic acid (ester of caffeic acid, RA) is a secondary metabolite and polyphenol present in many culinary plants, such as rosemary, mint, basil, and perilla, that presents various well-documented biological effects, such as anti-inflammatory, antioxidant, antidiabetic, and antitumor properties. Despite the high therapeutic potential of RA, its intrinsic properties of poor water solubility and low bioavailability have limited its translation to clinical settings (Chung et al., 2020). Based on the low bioavailability and bioactivity of the abovementioned compounds, a putative reconstitution (combinatory chemistry) of RA, Lut, and RS could enhance the bioavailability and efficiency to achieve maximal response against the limitations. This combination is worth investigating as it can potentially help lower blood glucose levels in diabetes.

In recent times, researchers have been keen on developing methods to induce antihyperglycemia by eliminating starch digestion, promoting metabolism (glucose uptake, absorption, and utilization), and triggering insulin sensitivity; approaches have been employed to explore good metabolite candidates and further determine the best combination ratio for phytotherapeutics. In this study, the best or gold combination of known antidiabetic metabolites, specially RA, Lut, and RS, was tailored on the basis of IC_50_, solubility, and network pharmacology studies. Then, molecular docking of these selected metabolites was examined toward diabetic multitarget proteins, such as α-amylase, α-glucosidase, pancreatic lipase, SGLT-2, AMPK, glucokinase, aldose reductase, acetylcholinesterase, acetylcholine M2 receptor, GLP-1R, DPP-IV, and PPAR-γ, which are extensively considered therapeutic targets in clinical treatments for maximal response to reducing blood glucose levels. Furthermore, *in vitro* α-amylase inhibition assays with the lone metabolites and combination as well as *in vivo* tests (oral starch tolerance test, OSTT, and oral glucose tolerance test, OGTT) in diet-induced obese diabetic mice were conducted to verify the combination for putative antidiabetic effects. Finally, the molecular mechanism of the combination was predicted through a network pharmacology study.

## 2 Materials and methods

### 2.1 Materials

Purified Lut (98% purity), RS (98%), and RA (98%) were obtained from Shaanxi Dongshuo Biotechnology Co., Ltd. (Shaanxi, China).

### 2.2 Pharmacokinetics and ADME/toxicity profiling

The pharmacokinetic properties of the materials, such as ADMET behaviors of the ligands in the human body, were screened using the SwissADME (http://www.swissadme.ch/index.php) and admetSAR prediction tool webserver (http://lmmd.ecust.edu.cn/admetsar2). This step is significant for identifying the drug likeness, medicinal chemistry, lead likeness, and toxicity potential of new candidate drugs, phytochemicals, food additives, and industrial chemicals; it is also a prerequisite for establishing valid complementary methods before *in vivo/in vitro* analyses (Cheng et al., 2012; Sharma et al., 2020; Suchitra et al., 2020).

### 2.3 Exploring the potential targets of RA, Lut, and RS

The Traditional Chinese Medicine Systems Pharmacology Database and Analysis Platform (TCMSP) (https://tcmsp-e.com/) (Liu et al., 2013) were used to obtain the potential targets of RA, Lut, and RS (RLR). The target names were standardized using the UniProt database (https://www.uniprot.org/) for the status criterion of “Reviewed” and organism category of “Human”.

### 2.4 Acquisition of diabetic hyperglycemia and T2DM related targets as well as construction of Venn diagrams

Diabetic hyperglycemia targets were screened using the DrugBank database; further, the RCSB database was searched for the target protein database (PDB) using qualifiers such as “*Homo sapiens*,” “X-ray,” and “no mutation.” These targets were compared with the RLR targets, and Venn diagrams were constructed to identify the targets related to both diabetic hyperglycemia and RLR. T2DM-related targets were obtained from four databases, namely, the Comparative Toxicogenomics Database (CTD) (http://ctdbase.org/), GeneCards (https://www.genecards.org/), OMIM (https://www.omim.org), and DisGeNET (https://www.disgenet.org/). Threshold scores were simultaneously set in the CTD, GeneCards, and DisGeNET to filter the targets; all targets from the four databases were merged, and duplicate values were deleted to obtain the corresponding T2DM targets. Venn diagrams were also constructed for the T2DM targets using the tools in Hiplot Pro (https://hiplot.com.cn/), a comprehensive web service for biomedical data analysis and visualization. The common targets between RLR and T2DM (i.e., T2DM-related targets treated by RLR) were obtained from the Venn diagrams.

### 2.5 Construction of regulatory networks between RLR and the intersecting targets

Cytoscape (version 3.9.1; https://cytoscape.org/) (Shannon et al., 2003) was used to visualize the interactions of RLR with diabetic hyperglycemia and T2DM as a regulatory network. The intersecting targets and different types of metabolites were displayed using various shapes. The degree value represents the number of interactions generated by a node, and a higher degree value denotes a more significant status in the network. These higher degree values were represented using deeper colors.

### 2.6 Network analysis of protein–protein interactions (PPIs) of the intersecting targets

The intersecting targets were imported into the STRING 11.0 platform and visualized using Cytoscape 3.9.1 software to construct the PPI network. The CytoHubba plugin was applied to analyze and obtain the top-10 hub genes ranked by degree value (Chin et al., 2014). The MCODE plugin was then used to analyze the most significant module and determine the top-10 hub targets ranked on the basis of the MCODE score in the module (Saito et al., 2012). The K-means clustering algorithm was used with the MCODE plugin, and the module with the highest score was considered to be the most significant module. Nodes with higher MCODE scores were assigned more significant statuses in the general PPI network.

### 2.7 GO and KEGG pathway enrichment analyses

The R packages “clusterProfiler,” “org.Hs.eg.db,” “ggplot2,” and “DOSE” were used to perform gene ontology (GO) and Kyoto encyclopedia of genes and genomes (KEGG) pathway enrichment analyses, and the results were visually displayed using R 4.2.3 software. The statistical significance for the enrichment analysis was an adjusted p value ≤0.05. Three aspects of the GO analysis, namely, molecular function (MF), biological process (BP), and cellular component (CC), which were most significantly associated with the top-10 GO functional terms were selected in each field. Correspondingly, the KEGG pathway enrichment analysis was conducted to investigate the intersecting genes, and results were obtained for the top-20 pathways.

### 2.8 Molecular docking

All structures of the tested target proteins were first downloaded from the RCSB repository with PDB numbers (https://www.rcsb.org/), namely, α-amylase (5U3A; 4GQR), α-glucosidase (3TOP; 3L4Y), pancreatic lipase (1LPA), SGLT-2 (7VSI), AMPK (6C9F), glucokinase (3A0I), aldose reductase (1IEI), acetylcholinesterase (4BDT), acetylcholine M2 receptor (4MQT), GLP-1R (7C2E), DPP-IV (4N8D), and PPAR-γ (1WM0; 4CI5). The criteria for target selection were as follows: “*H. sapiens*” and “no mutation.” The key residues used as constituents of the putative binding pockets were found from the corresponding literature in the RCSB repository based on PDB numbers. For each protein structure, the selected key residues were applied to constitute the putative binding pocket (Supplementary Table S1 footnotes)**.**

Originally, the ligands and water molecules were removed from the protein crystal, to which hydrogen and the desired electric charge were added using Discovery Studio 2019. Next, the protein structures were subjected to energy minimization before docking. In the Simulation | Change Forcefield tools, CHARMM36 was one of the versions applied to minimize the energy. Second, the test chemicals (ligands) were sourced from PubChem (https://pubchem.ncbi.nlm.nih.gov/; RA (5281792), Lut (5280445), and RS (445154)) and were also charged with hydrogen atoms. Finally, molecular simulations were performed, whose results showed that the ligand was located inside the grid box of the receptor. The lowest binding energy (kcal/mol) was calculated using AutoDock Vina 1.2.0, and the 2D and 3D images were visualized and analyzed using Discovery Studio 2019.

### 2.9 α-Amylase activity assay

The α-amylase inhibitory evaluations of the selected metabolites (RA, Lut, and RS) were performed using the 3,5 dinitrosalicylic acid (DNSA) colorimetric assay (Chen T.-H. et al., 2021; Chen S.-P. et al., 2021). The α-amylase activity was quantified based on the reduction of sugars from the breakdown of starch, using DNSA dissolved in 2 M NaOH/5.3 M Na^+^-K^+^-tartaric acid, as previously described (Luyen et al., 2018). In brief, the substrate solution was prepared by dissolving starch (4 mg/mL) in 20 mM of phosphate-buffered saline (PBS 1×, pH 6.8). Approximately 50 μL of each sample solution (various concentrations in each of the test chemicals) and 100 μL of 16 unit/mL α-amylase were added to 1.5-mL Eppendorf tubes and incubated for 10 min. Next, 100 μL of the substrate solution was added to each mixture and incubated at 37°C for an additional 30 min. Finally, 50 μL of the DNS reagent was added to each mixture and boiled for 10 min. The optical densities of the samples were detected at 540 nm (OD_540_) using the Infinite^®^ M200 PRO multimode microplate reader (Tecan, Switzerland) with acarbose as the positive control.

### 2.10 Diet-induced diabetic model preparation

#### 2.10.1 Animal care and diet-induced obesity induction

Sixty male ICR mice (6 weeks old) were obtained from Wu’s Laboratory Animals (Fujian, China) and housed in controlled environmental conditions at room temperature (22°C ± 2°C) and humidity (50% ± 10%). A 12/12 h light/dark (6 a.m. to 6 p.m.) cycle was maintained throughout the study period. The mice had free access to food as well as tap water and were maintained on a standard laboratory diet (Rodent feed 1022, BEIJING HFK BIOSCIENCE Co., Ltd., Beijing, China). The animal experiments were approved by the Xiamen Medical College Animal Ethics Committee (SYXK, 2018-0010) and were conducted in accordance with the “Guide for the Care and Use of Laboratory Animals” of Xiamen Medical College.

#### 2.10.2 Insulin intolerance test (IGT) and determination of diabetic mice

The protocols for the diet-induced obese (DIO) mice and glucose tolerance (GT) were followed as per our previous study (Huang et al., 2019a). First, DIO mice were fasted for 10 h prior to oral gavage of 4 g/kg bodyweight (Bwt) glucose solution. At the beginning of the test, the fasting blood glucose levels of the mice were measured from tail-vein samples using an Accu-Chek blood glucose analyzer (Hoffmann-La Roche AG, Basel, Switzerland).

#### 2.10.3 OGTT

The OGTT of the diabetic mice were performed as per the procedures described in a previous report (Huang et al., 2019a), with slight modifications. For this test, a total of 48 DIO diabetic mice were divided into eight groups (n = 6) as control (water), positive control (125 mg/kg Bwt of metformin dissolved in water), Lut (12.5 mg/kg Bwt dissolved in edible oil), RA (66.0 mg/kg Bwt dissolved in edible oil), RS (91.0 mg/kg Bwt dissolved in edible oil), RLR mixture (RA: Lut: RS = 5:1:4; fasting blood glucose >7 mmol/L; 100 mg/kg Bwt), RLR-H (fasting blood glucose >10 mmol/L; 100 mg/kg Bwt), and insulin (0.2 U/kg intraperitoneally). Then, 4 g/kg Bwt of glucose solution (fresh preparation, dissolved in water) was provided during the test at various times (30, 60, 90, and 120 min). The blood glucose levels of the mice were lastly measured from tail-vein samples using a blood analyzer.

#### 2.10.4 OSTT

For this test, a total of 24 DIO diabetic mice were divided into four groups (n = 6) as control (water only), positive control (10 mg/kg acarbose dissolved in water), RLR mixture (fasting blood glucose >7 mmol/L; 100 mg/kg Bwt), and RLR-H (fasting blood glucose >10 mmol/L; 100 mg/kg Bwt). The protocols for the OSTT were the same as those for OGTT but with three modifications: a) 3 g/kg Bwt of corn starch solution (fresh preparation, dissolved in water) was administered; b) the positive control used was acarbose (10 mg/kg); c) the test time was extended to 180 min. The blood glucose levels of the mice were again measured from tail-vein samples using a blood analyzer.

### 2.11 Statistical analysis

The data were expressed in terms of means ± SEM for the *in vivo* results and means ± SD for all other cases. Statistical comparisons of the results were conducted using one-way analysis of variance (ANOVA). The means in each column followed by different letters indicate the significant differences at *p* < 0.05 based on the *post hoc* Tukey’s test.

## 3 Results

### 3.1 ADMET

The *in silico* ADMET profiling of metabolites characteristically illustrates the latent to be profitable interactions between the potential drug candidates with specific protein targets for successful drug discovery and development. In this study, the deviations of RA, Lut, and RS are all within acceptable ranges. It was found that two of the chemicals, Lut and RA, behaved as HOB+, while RS behaved as HOB-. Therefore, the drug candidates reflect this blood–brain barrier (BBB) crossing with a topological polar surface area (TPSA) values as follows: RS <60.7 Å_2_; RA, Lut >90 Å_2_. All of the WLogP values were also found to be less than 6 (Ishola et al., 2021). Thus, the three phytochemical candidates examined in this study (RA, Lut, and RS) can cause transversion of the BBB; however, two of the candidates exhibit carcinogenic properties. The BBB is essential for restricting in/outflux to the CNS microenvironment to ensure adequate neuronal function (Ishola et al., 2020). Although RA, Lut, and RS possess considerable binding affinities to the three antidiabetic protein targets, their significant BBB transversion could be employed to develop drugs against neurodegenerative diseases. Caco2 cells are widely used as a model of the intestinal epithelial cells exposed to the intestinal lumen, which is the location of action for pancreatic α-amylase and α-glucosidase (Lea, 2015). Furthermore, all three candidates are readily absorbed at the intestine and are negative. The accessibility of a drug candidate through a membrane is determined by its Caco2 permeation, and this attribute is especially notable in the case of RA, Lut, and RS. These three phytochemicals as potential drug candidates therefore substantially pass the profiling tests (Table 1). We assessed the bioavailability and toxicity of the metabolites using Lipinski’s rule-of-five and ADMET analysis, and the metabolites fulfil all the listed criteria, similar to the findings of a previous study (Sharma et al., 2020), suggesting their suitability for the development of potent antidiabetic drugs.

**TABLE 1:** **(A)** Physicochemical properties and **(B)** *in silico* ADME/toxicity profiles of rosmarinic acid, luteolin, and resveratrol.

Property name	Luteolin	Resveratrol	Rosmarinic acid
(A) Physicochemical properties			
Molecular weight	**286.24**	**228.24**	**360.3**
XLogP3	**1.4**	**3.1**	**2.4**
Hydrogen bond donor count	**4**	**3**	**5**
Hydrogen bond acceptor count	**6**	**3**	**8**
Rotatable bond count	**1**	**2**	**7**
Topological polar surface area (TPSA)	**107 Å** ^**2**^	**60.7 Å** ^**2**^	**145 Å** ^**2**^
Heavy atom count	**21**	**17**	**26**
Formal charge	**0**	**0**	**0**
Complexity	**447**	**246**	**519**
Defined atom stereocenter count	**0**	**0**	**1**
Defined bond stereocenter count	**0**	**1**	**1**
Covalently bonded unit count	**1**	**1**	**1**
Compound is canonicalized	**Yes**	**Yes**	**Yes**
(B) ADMET			
Human intestinal absorption	**HIA-**	**HIA+**	**HIA-**
Human oral bioavailability	**HOB+**	**HOB-**	**HOB+**
Blood–brain barrier	**BBB+**	**BBB+**	**BBB+**
Caco2 permeability	**Caco2+**	**Caco2+**	**Caco2+**
Acute oral tox log (1 mol/kg)	**Nil**	**Nil**	**Nil**
Carcinogenic	**-**	**-**	**-**
CYP2C9	**-**	**+**	**-**
CYP2D6	**+**	**-**	**-**
CYP1A2	**+**	**+**	**-**
CYP2C19	**-**	**-**	**-**
CYP3A4	**+**	**+**	**-**
Hepatotoxicity	**-**	**-**	**-**
Lipinski rule violation	**Nil**	**Nil**	**Nil**
Lead likeness violation	**Nil**	**1**	**1**
Solubility LogS	**−2.588**	**−2.439**	**−3.154**

Footnote: ADMET, Absorption, Distribution, Metabolism, Excretion, Toxicity; +, positive; -, negative; solubility normal range: −6.5 to 0.5.

HIA% < 30% = HIA-; HIA% > 30% = HIA+; CYP2C9 inhibitor, CYP2D6 inhibitor, CYP1A2 inhibitor, CYP2C19 inhibitor.

### 3.2 IC_50_ and solubility

Based on a review of the half-maximal inhibitory concentrations (IC_50_) from literature, the solubility and IC_50_ (*in vitro* and *in vivo*) are determined for the various proteins of RA, Lut, and RS (Table 2). Based on the retrieved IC_50_ and solubility values, the ratio of RA: Lut: RS is determined to be 5:1:4 for the subsequent experiments**.**

**TABLE 2:** Solubility and IC_50_ (*in vitro* and *in vivo*) values of rosmarinic acid, luteolin, and resveratrol. DMSO: dimethyl sulfoxide, DMF: dimethyl formamide.

Ligands	Solubility	IC_50_ (μM)						EC_50_ (μM)	
		α-Amylase^1–4^	α-Glucosidase^2,5–9^	DPP-IV^2,10^	PTP-1B^11,12^	Aldose reductase^13,14^	Pancreatic lipase^15,16^	SIRT1^17^	PPAR-γ^18^
Luteolin	In methanol and alkaline solutions; slightly in water, DMSO (57 mg/mL), and ethanol (6 mg/mL)	147–360	26.41–172	0.12	136.3	0.6	63	-	2.3
C_15_H_10_O_6_									
CID: 5280445									
Resveratrol	In water (3 mg/100 mL); in ethanol, DMSO, and DMF (65 mg/mL)	32.23	47.93–123	5.638	-	117.6	-	7	-
C_14_H_12_O_3_									
CID: 445154									
Rosmarinic acid	In ethanol, DMSO, and DMF (25 mg/mL)	103	33	-	137	11.2	51.28	-	-
C_18_H_16_O_8_									
CID: 5281792									

References: 1. Kusano G, Takahira M, Shibano M, et al. Studies on inhibitory activities of fukiic acid esters on germination, alpha-amylase and carboxypeptidase A. *Biol Pharm Bull*. 1998; 21(9):997-999. doi: 10.1248/bpb.21.997.

2. Khalid MF, Rehman K, Irshad K, Chohan TA, Akash MSH. Biochemical investigation of inhibitory activities of plant-derived bioactive compounds against carbohydrate and glucagon-like peptide-1 metabolizing enzymes. *Dose Resp*. 2022; 20(2):15593258221093276. doi: 10.1177/15593258221093275.

3. Yang Y, Wang Y, Zeng W, et al. A strategy based on liquid-liquid-refining extraction and high-speed counter-current chromatography for the bioassay-guided separation of active compound from *Taraxacum mongolicum*. *J Chromatogr A*. 2020; 1614:460727. doi: 10.1016/j.chroma.2019.460727.

4. Tadera K, Minami Y, Takamatsu K, Matsuoka T. Inhibition of alpha-glucosidase and alpha-amylase by flavonoids. *J Nutr Sci Vitaminol (Tokyo)*. 2006; 52(2):149-153. doi: 10.3177/jnsv.52.149.

5. Yan J, Zhang G, Pan J, Wang Y. α-Glucosidase inhibition by luteolin: Kinetics, interaction and molecular docking. *Int J Biol Macromol*. 2014; 64:213-223. doi: 10.1016/j.ijbiomac.2013.12.007.

6. Wagle A, Seong SH, Shrestha S, Jung HA, Choi JS. Korean thistle (*Cirsium japonicum* var. maackii (Maxim.) Matsum.): A potential dietary supplement against diabetes and Alzheimer’s disease. *Molecules*. 2019; 24(3):E649. doi: 10.3390/molecules24030649.

7. Kubínová R, Pořízková R, Navrátilová A, et al. Antimicrobial and enzyme inhibitory activities of the constituents of *Plectranthus madagascariensis* (Pers.) Benth. *J Enzyme Inhib Med Chem*. 2014; 29(5):749-752. doi: 10.3109/14756366.2013.848204.

8. Ablat A, Halabi MF, Mohamad J, et al. Antidiabetic effects of *Brucea javanica* seeds in type 2 diabetic rats. *BMC Complement Altern Med*. 2017; 17(1):94. doi: 10.1186/s12906-017-1610-x.

9. Zhang CC, Geng CA, Huang XY, Zhang XM, Chen JJ. Antidiabetic stilbenes from peony seeds with PTP1B, α-glucosidase, and DPPIV inhibitory activities. *J Agri Food Chem*. 2019; 67(24):6765-6772. doi: 10.1021/acs.jafc.9b01193.

10. Fan J, Johnson MH, Lila MA, Yousef G, de Mejia EG. Berry and citrus phenolic compounds inhibit dipeptidyl peptidase IV: Implications in diabetes management. *Evid Based Complement Altern Med*. 2013; 2013:479505. doi: 10.1155/2013/479505.

11. Huang Q, Chen JJ, Pan Y, et al. Chemical profiling and antidiabetic potency of *Paeonia delavay*i: Comparison between different parts and constituents. *J Pharm Biomed Anal*. 2021; 198:113998. doi: 10.1016/j.jpba.2021.113998.

12. Salinas-Arellano E, Pérez-Vásquez A, Rivero-Cruz I, et al. Flavonoids and terpenoids with PTP-1B inhibitory properties from the infusion of *Salvia amarissima Ortega*. *Molecules*. 2020; 25(15):E3530. doi: 10.3390/molecules25153530.

13. Kim SB, Hwang SH, Wang Z, Yu JM, Lim SS. Rapid identification and isolation of inhibitors of rat lens aldose reductase and antioxidant in *Maackia amurensis*. *Biomed Res Int*. 2017; 2017:4941825. doi: 10.1155/2017/4941825.

14. Ha TJ, Lee JH, Lee MH, et al. Isolation and identification of phenolic compounds from the seeds of *Perilla frutescens* (L.) and their inhibitory activities against α-glucosidase and aldose reductase. *Food Chem*. 2012; 135(3):1397-1403. doi: 10.1016/j.foodchem.2012.05.104.

15. Afifi FU, Kasabri V, Litescu S, Abaza IF, Tawaha K. Phytochemical and biological evaluations of *Arum hygrophilum Boiss*. (Araceae). *Pharmacogn Mag*. 2017; 13(50):275-280. doi: 10.4103/0973-1296.204551.

16. Ramirez G, Zamilpa A, Zavala M, Perez J, Morales D, Tortoriello J. Chrysoeriol and other polyphenols from *Tecoma stans* with lipase inhibitory activity. *J Ethnopharmacol*. 2016; 185:1-8. doi: 10.1016/j.jep.2016.03.014.

17. Sharma S, Misra CS, Arumugam S, et al. Antidiabetic activity of resveratrol, a known SIRT1 activator in a genetic model for type-2 diabetes. *Phytother Res*. 2011; 25(1):67-73. doi: 10.1002/ptr.3221.

18. Liu Q, Yang QM, Hu HJ, et al. Bioactive diterpenoids and flavonoids from the aerial parts of *Scoparia dulcis*. *J Nat Prod*. 2014; 77(7):1594-1600. doi: 10.1021/np500150f.

### 3.3 Acquisition of the targets of RLR, diabetic hyperglycemia, and T2DM

The screening results from the TCMSP provided 146 targets of RS, 54 of Lut, 32 of RA, and 186 targets in total (Figure 3A). There were 43 related targets of diabetic hyperglycemia, as screened from the DrugBank database. The screening of T2DM targets showed 937 from the CTD, 336 from GeneCards, 247 from DisGeNET, and 39 from OMIM (Figure 3B). Pathway annotations and component–target–interaction (CTI) network constructions were performed for the subsequent assays.

### 3.4 Metabolite–target–pathway network analysis between active targets of diabetic hyperglycemia and RLR

**FIGURE 1:**
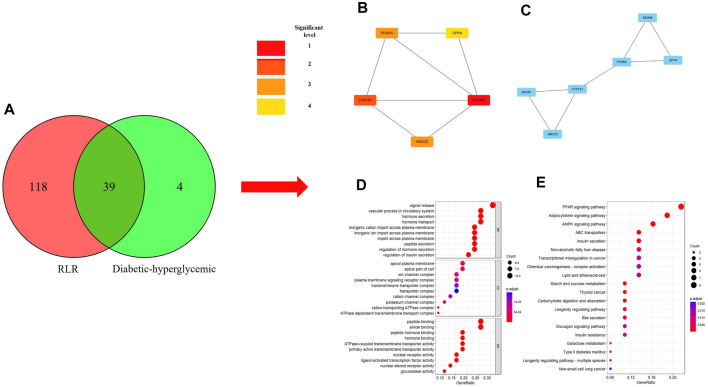
**(A)** Intersecting targets between rosmarinic acid, luteolin, and resveratrol (RLR) and diabetic hyperglycemia. **(B)** PPI network analysis. Nodes with red labels are the top-5 hub targets as analyzed by Cytohubba, and darker node colors represent higher scores. **(C)** Blue nodes represent nodes in the most crucial module analyzed by MCODE. **(D)** GO enrichment analysis of 39 intersecting targets. The top-10 GO functional terms were selected finally. **(E)** KEGG pathway enrichment analysis of 39 intersecting targets. The top-20 pathways were identified (adjusted p ≤0.05). The colors represent the adjusted p values, and spot sizes represent the gene counts.

**FIGURE 2:**
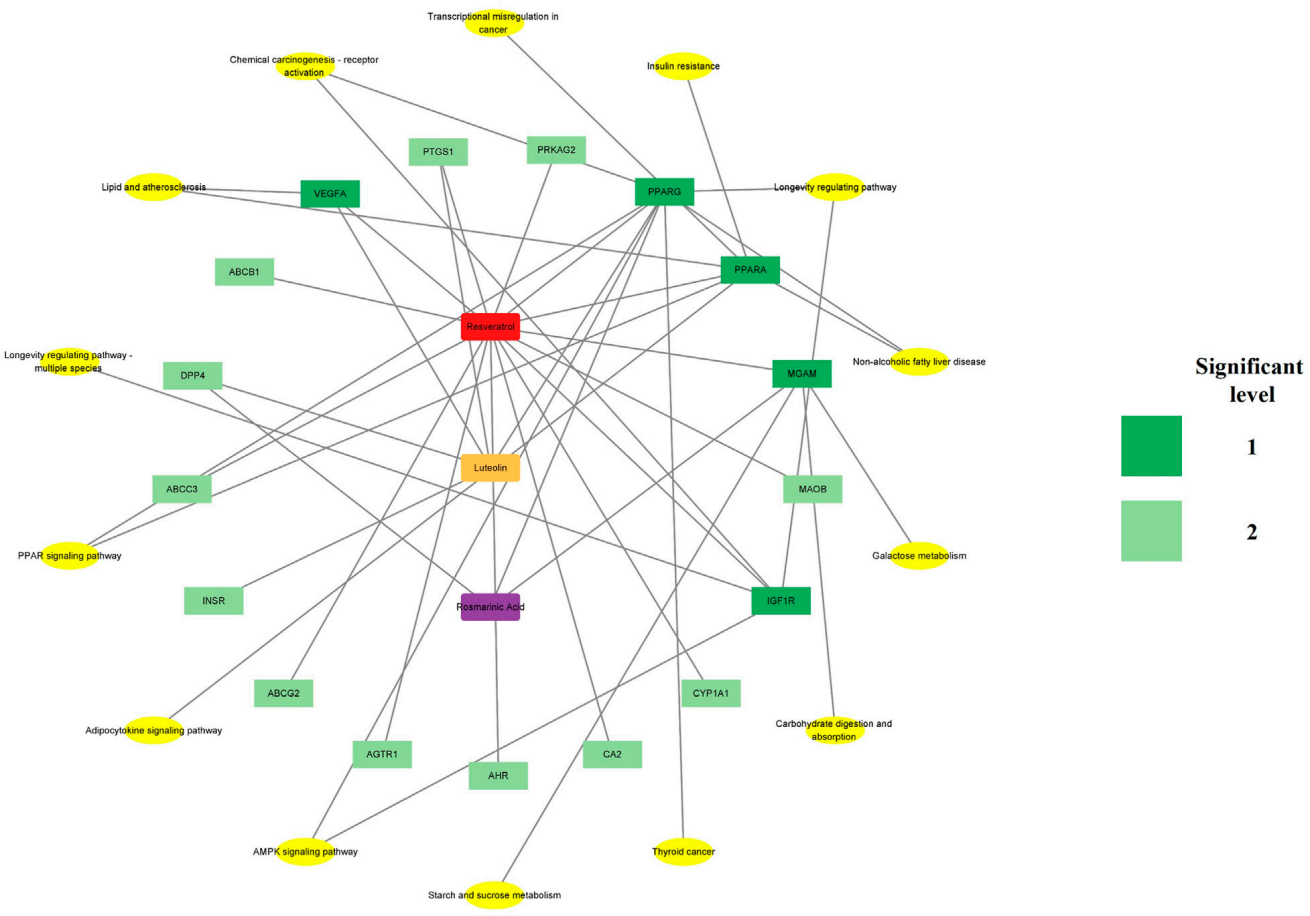
Metabolite–target–pathway network of rosmarinic acid (RA), luteolin (Lut), and resveratrol (RS) (RLR) showing the diabetic–hypoglycemic effects. The red, orange, and purple nodes represent RS, Lut, and RA, respectively. The dark and light green colors indicate the significance of the targets for the related core pathways, and the yellow nodes represent the pathways.

Cytoscape 3.9.1 was used to construct the PPI and CTI networks. Venn diagrams of the related targets were used for the PPI analysis as well as GO and KEGG pathway enrichment analyses (Figure 1A). The PPI network comprised 13 nodes and 25 edges. The top-5 hub targets in Cytohubba ranked by degree value were PPARG, ABCG2, CYP1A1, PPARA, and DPP4. The top-5 hub targets ranked by MCODE score were PPARA, MGAM, ABCC3, CYP1A1, and DPP4. The PPI results showed that PPARA and DPP4 were critical genes based on the two algorithms (Figures 1B, C). The KEGG and GO enrichment analyses showed that the core signaling pathway was the PPAR signaling pathway (Figures 1D, E). To identify the relationship between RLR and diabetic hyperglycemia, a metabolite–target–pathway network diagram was constructed using the results of the above analyses (Figure 2).

### 3.5 Intersection between RLR- and T2DM-related targets

**FIGURE 3:**
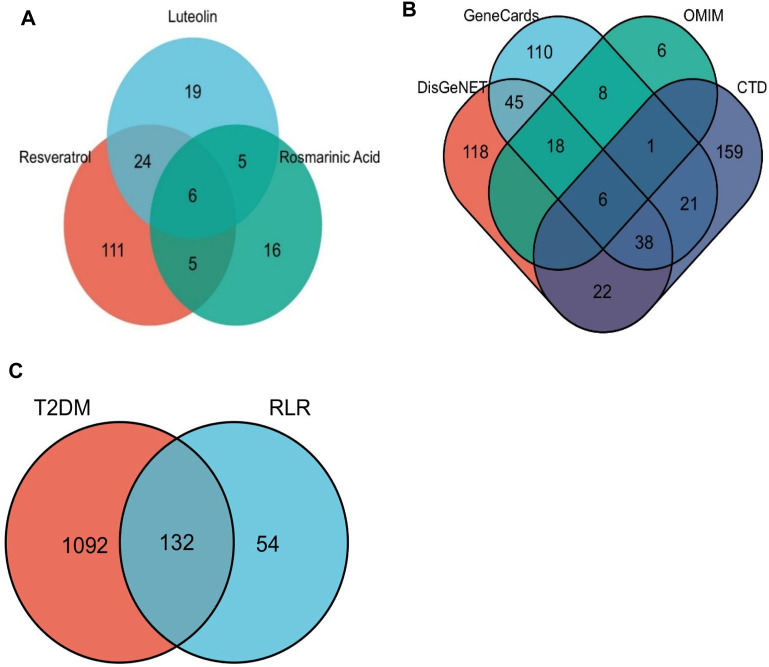
Venn diagrams of **(A)** rosmarinic acid, luteolin, and resveratrol (RLR) targets from TCMSP. **(B)** T2DM-related targets obtained from four databases. **(C)** Intersecting targets between T2DM and RLR.

A Venn diagram was used to obtain 132 intersecting targets between RLR and T2DM, as shown in Figure 3C. Accordingly, all three candidates (RA, Lut, and RS) have good links with the T2DM targets.

### 3.6 Establishment and analysis of CTI network between RLR and intersecting targets

**FIGURE 4:**
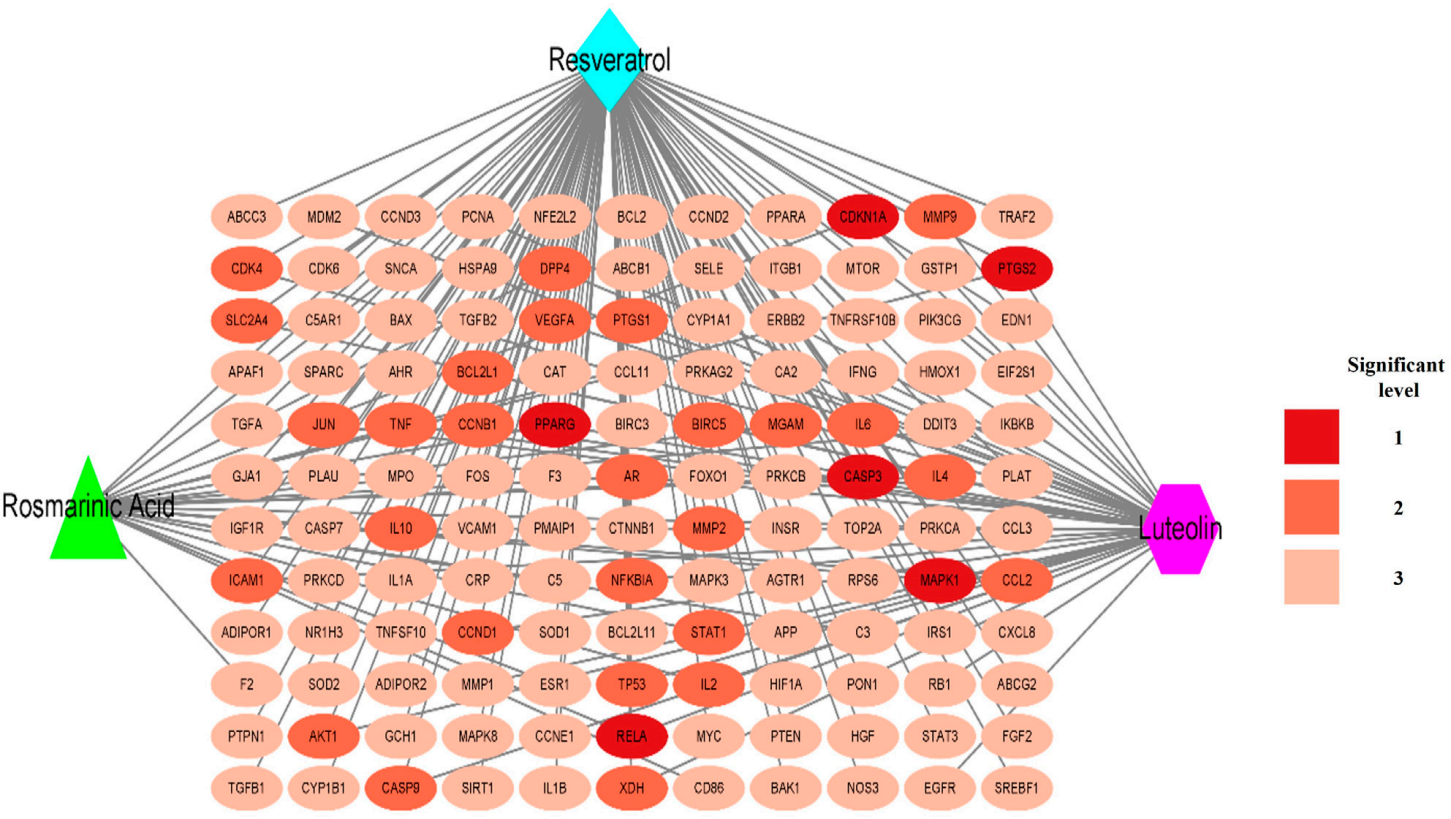
Establishment of the component–target–interaction network. The red elliptical nodes represent the intersecting targets, while the blue diamond, green triangle, and purple hexagon nodes represent rosmarinic acid, luteolin, and resveratrol (RLR), respectively. The connecting lines represent the interactions between the metabolites and targets.

The CTI network (Cytoscape 3.9.1) encompasses 135 nodes and 171 edges. As shown in Figure 4, all three components (RLR) had broad interactions with the corresponding targets of T2DM that are treatable by RLR. RS had 104, Lut had 44, and RA had 23 interactions with the intersecting targets. The degree values of the intersecting targets range from 1 to 3 with a red-colored gradient. According to the CTI results, PPARG, CDKN1A, PTGS2, CASP3, MAPK1, and RELA had interactions with the three components (RLR).

### 3.7 Prediction of RLR hub targets using PPI network of the intersecting targets

**FIGURE 5:**
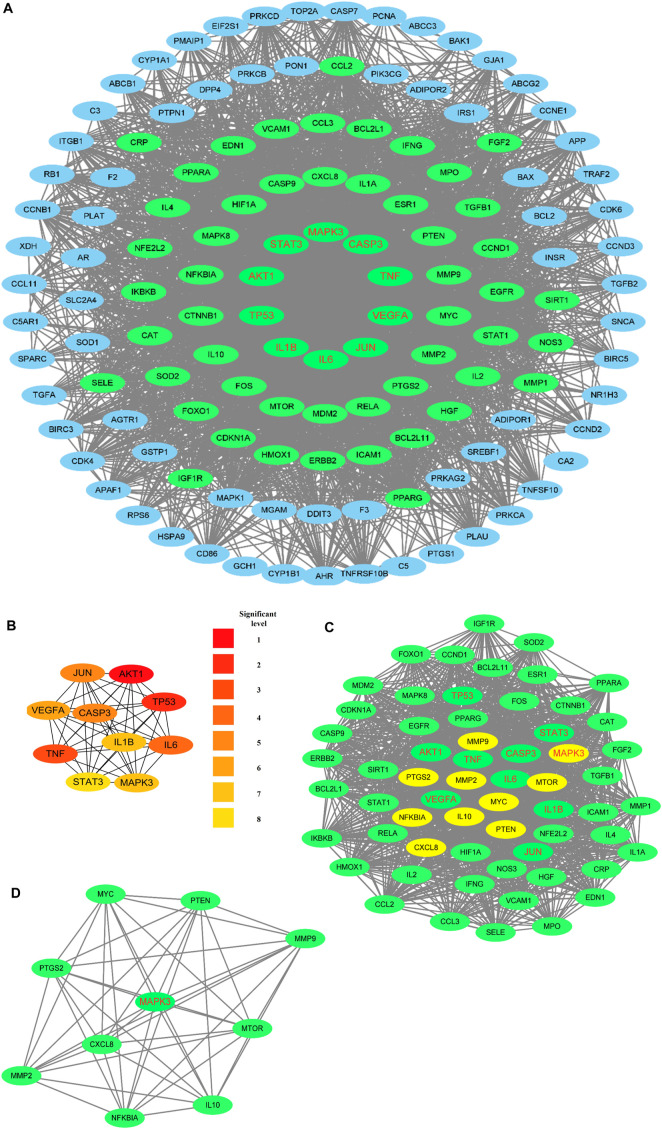
PPI networks: nodes with red labels are the top-10 hub targets obtained using Cytohubba, and the green nodes represent the nodes in the most crucial module analyzed using MCODE. **(A)** PPI network of T2DM-related targets that can be treated by rosmarinic acid, luteolin, and resveratrol (RLR). **(B)** Top-10 hub targets screened using CytoHubba, where the darker node colors represent higher scores. **(C)** Analysis of the most significant module using MCODE, where the yellow nodes represent the top-10 hub targets ranked on the basis of the MCODE score. **(D)** Top-10 hub targets ranked by MCODE score.

The PPI network comprised 132 nodes and 3140 edges (Figure 5A). The top-10 hub targets in Cytohubba ranked by degree value were AKT1, TP53, TNF, IL6, CASP3, JUN, VEGFA, IL1B, MAPK3, and STAT3 (Figure 5B). The most significant cluster with a score of 49.97 contained 61 nodes and 1499 edges, as obtained from MCODE analysis (Figure 5C). The top-10 hub targets ranked by MCODE score were NFKBIA, IL10, CXCL8, MMP9, MMP2, PTGS2, MAPK3, MYC, MTOR, and PTEN (Figure 5D). Remarkably, MAPK3 was found to be a critical gene under both methods.

### 3.8 Prediction of RLR molecular mechanism via GO and KEGG pathway enrichment analyses

**FIGURE 6:**
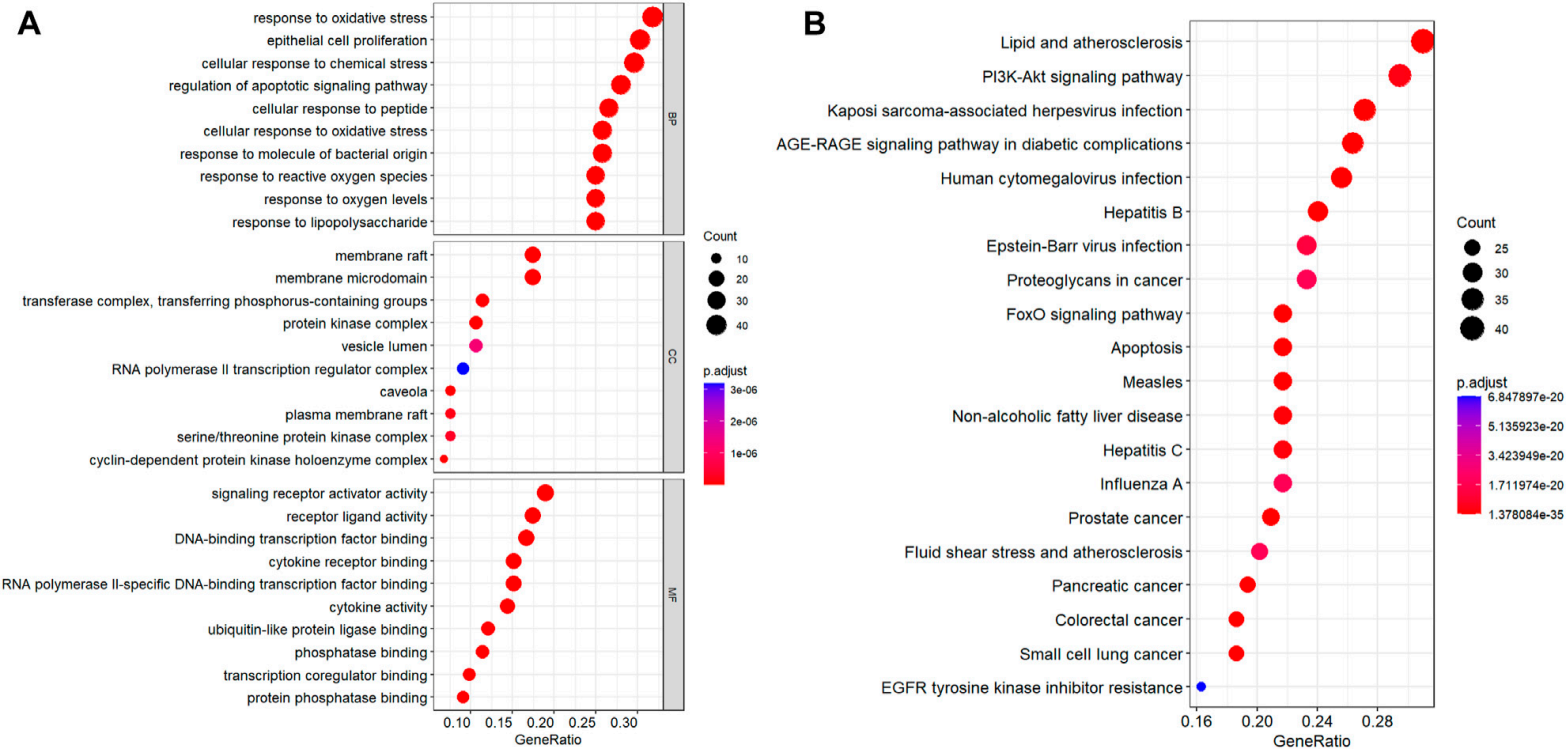
Color representations of the adjusted p scores and spot sizes indicating gene counts. **(A)** GO enrichment results of 132 intersecting targets, from which the top-10 GO functional terms were selected. **(B)** KEGG pathway enrichment results of 129 intersecting targets, from which the top-20 pathways were identified. (adjusted p ≤0.05).

**FIGURE 7:**
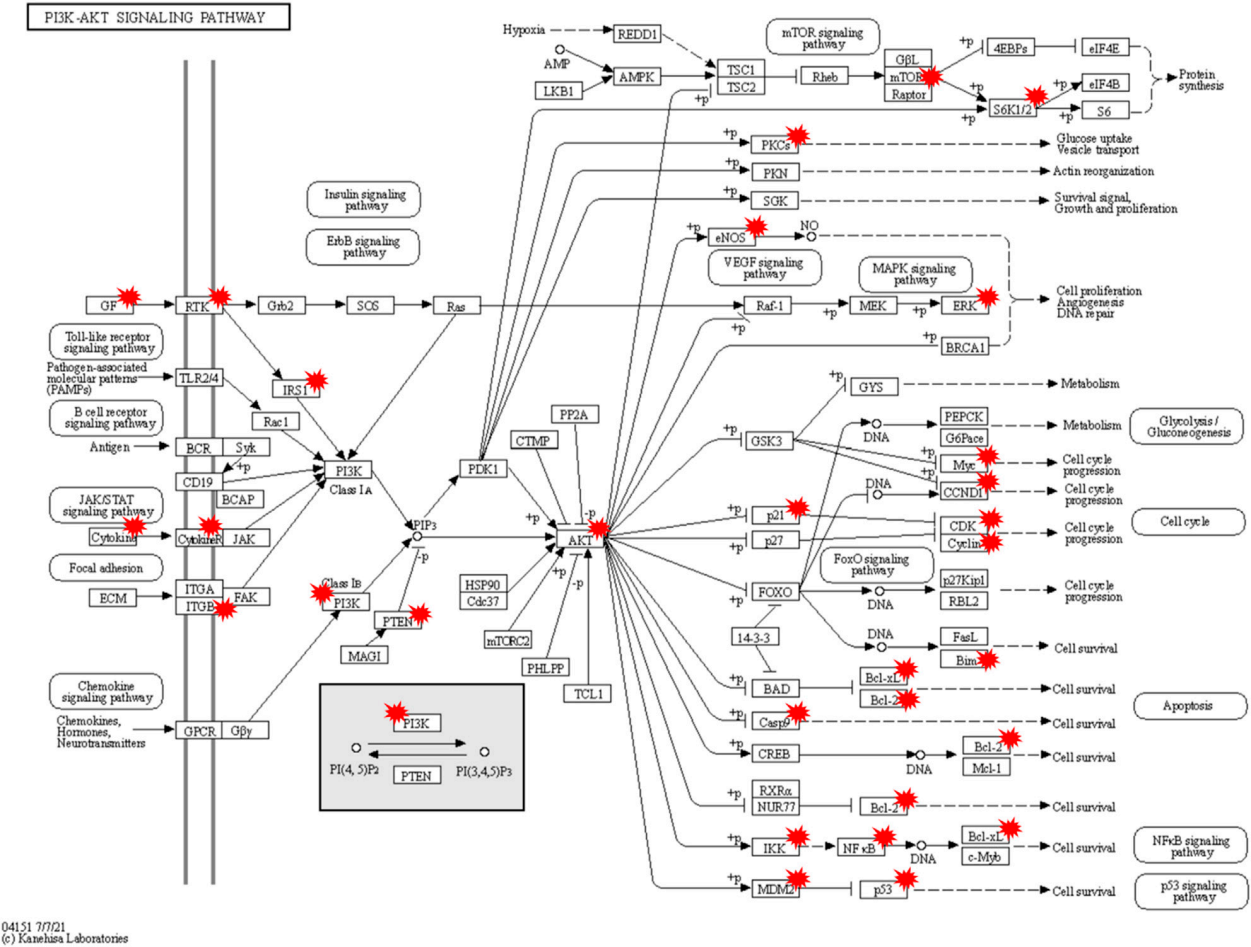
Potential molecular mechanisms of rosmarinic acid, luteolin, and resveratrol (RLR) in T2DM treatment via regulation of the PI3K-AKT signaling pathway. The targets with red markers can be treated by RLR.

A total of 132 intersecting targets were analyzed by GO enrichment, and the results comprised 2584 BPs, 50 CCs, and 201 MFs obtained using R studio (Figure 6A). When sorted by the degree of significance, the top-3 BPs were responses to oxidative stress, epithelial cell proliferations, and cellular responses to chemical stress; the top-3 CCs were membrane rafts, membrane microdomains, and transferase complexes transferring phosphorus-containing groups; the top-3 MFs were signaling receptor activator activity, receptor ligand activity, and DNA-binding transcription factor binding. Furthermore, 129 KEGG enrichment pathways related to T2DM were obtained, of which the top-30 signaling pathways of significance were selected for visual presentation (Figure 6B). Among the enriched pathways, the PI3K-AKT signaling pathway has great significance in T2DM treatment, and RLR acted on multiple targets of the PI3K-AKT signaling pathway, as shown in Figure 7.

### 3.9 *In silico* docking

Nowadays, the application of computational techniques offers several advantages in rationalizing the route toward the discovery of novel drugs or identification of the biological properties of compounds (both natural and synthetic origins) for use in the treatment or supplementation of several diseases (Gelmini et al., 2018; Grande et al., 2021). In particular, molecular docking allows fitting a ligand to a binding site by combining and optimizing the steric, hydrophobic, and electrostatic complementarity variables. Furthermore, one of the main goals of molecular docking is the identification of the most energetically favorable conformation of a ligand at the binding site of the target molecule (the more negative binding energy is the better ligand–target stability). An absolute value greater than 4.25 indicates certain binding activity, a value exceeding 5.0 indicates good binding activity, and a value exceeding 7.0 indicates strong binding activity (Hsin et al., 2013). To clarify the intermolecular affinity between the metabolites (RA, Lut, and RS) and specific targets, molecular docking simulations were performed. The docking analysis revealed that twelve therapeutic molecular targets, namely, α-amylase, α-glucosidase, pancreatic lipase, SGLT-2, AMPK, glucokinase, aldose reductase, acetylcholinesterase, acetylcholine M2 receptor, GLP-1R, DPP-IV, and PPAR-γ, showed good binding affinities and better binding modes (as shown in Supplementary Figure S1 of the Supplementary Material).

**FIGURE 8:**
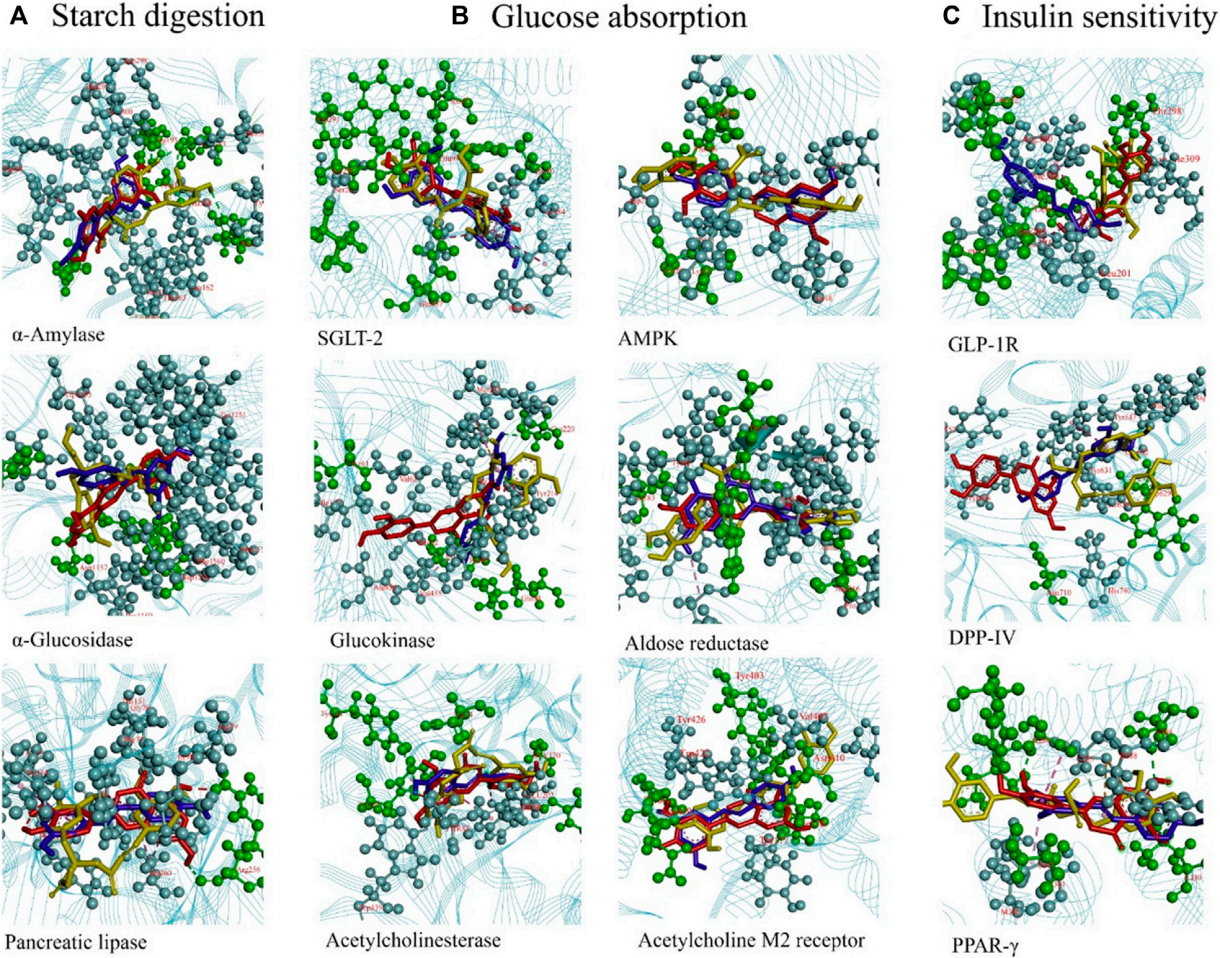
**(A)** Rosmarinic acid (RA), luteolin (Lut), and resveratrol (RS) binding with the diabetes-related proteins α-amylase, α-glucosidase, and pancreatic lipase to control starch digestion. **(B)** Lut, RA, and RS binding with proteins SGLT-2, AMPK, glucokinase, aldose reductase, acetylcholinesterase, and acetylcholine M2 receptor to mediate glucose absorption. **(C)** Lut, RA, and RS binding with proteins GLP-1R, DPP-IV, and PPAR-γ to regulate insulin sensitivity. RA: yellow; Lut: red; RS: blue.

Supplementary Table S1 and Figure 8A show the targets controlling starch digestion. The α-amylase (PDB: 5U3A) modeling results revealed that RA formed H-bonds with ARG195, GLU233, and HIS201; Lut formed H-bonds with GLN63, ASP197, and GLU233; and RS formed H-bonds with ASP197. The reference drug acarbose is a catalytic inhibitor that formed H-bonds with GLU233, HIS201, TRP58, and THR163. For α-glucosidase (PDB: 3TOP), RA had H-bonds with ASP1526, TRP1369, ARG1510, and ASP1420; Lut had H-bonds with ASP1526; and RS had H-bonds with ASP1526. The H-bond interactions of RA and RS against α-glucosidase occur at the same amino acid residue (ASP1526). When comparing the binding affinities of five standard inhibitor drugs (acarbose, miglitol, voglibose, emiglitate. and 1-deoxynojirimycin) with three ligands (RA, Lut, RS) of α-glucosidase, Lut possessed the best binding affinity (−9.3 kcal/mol) out of the five standard inhibitor drugs. When compared with acarbose as the reference drug, the binding affinities of RA, Lut, and RS on α-amylase and α-glucosidase were higher (Lut > RA > RS > Acarbose), suggesting that they can be used as competitive inhibitors. For pancreatic lipase (PDB: 1GPL), RA did not form any H-bonds with amino acid residues, while Lut formed H-bonds with ARG256 and RS formed H-bonds with ARG256.

The targets involved in the mediation of glucose absorption are illustrated in Supplementary Table S1 and Figure 8B. For the sodium-dependent glucose transporter 2 (SGLT-2) (PDB:7VSI), RA formed H-bonds with ASN75, HIS80, GLU99, TYP290, TYP291, and GLN457; Lut formed H-bonds with SER286; and RS formed H-bonds with ASN75. For adenosine 5’-monophosphate (AMP)-activated protein kinase (AMPK; PDB: 6C9F), RA formed H-bonds with THR106 and ASP108; Lut formed H-bonds with LEU20; and RS did not form any H-bonds. For glucokinase (PDB: 3A0I), RA formed H-bonds with GLN98; Lut formed H-bonds with TYR61; and RS formed H-bonds with CYS220, GLN98, and LEU451 amino acid residues. For aldose reductase (PDB: 1IEI), RA formed H-bonds with SER210, TRP20, and THR19; Lut formed H-bonds with ASP216 and GLN183; and RS formed H-bonds with GLN183 amino acid residue. For acetylcholinesterase (PDB: 4BDT), RA formed H-bonds with TYR337, ASP74, THR283, GLY120, and TYR133; Lut formed H-bonds with TYR341 and ASP74; and RS formed H-bonds with GLY120 and TYR341. For acetylcholine M2 receptor (PDB: 4MQT), RA did not form any H-bonds; Lut formed H-bonds with TYR80, ASN419, and ASN410; and RS formed H-bonds with LEU98 amino acid residue.

The targets involved in the regulation of insulin sensitivity are presented in Supplementary Table S1 and Figure 8C. The modeling results of the glucagon-like peptide-1 receptor (GLP-1R; PDB: 7C2E) revealed that RA formed H-bonds with ARG310 and THR298; Lut formed H-bonds with ARG310, LEU384, and THR298; and RS formed H-bonds with LEU32 and TYR298 amino acid residues. For dipeptidyl peptidase-4 (DPP-Ⅳ; PDB: 4N8D), RA formed H-bonds with ASP545, VAL546, TRP629, and TYR631; Lut formed H-bonds with TYP631 and ASN710; and RS formed H-bonds with TRP629 and VAL546 amino acid residues. For PPAR-γ (PDB: 1WM0), RA formed H-bonds with SER289, SER342, and GLY284; Lut formed H-bonds with HIS266, ILE281, and SER289; and RS formed H-bonds with LEU330. The 2D- and 3D-binding features of α-amylase, α-glucosidase, pancreatic lipase, SGLT-2, AMPK, glucokinase, aldose reductase, acetylcholinesterase, acetylcholine M2 receptor, GLP-1R, DPP-IV, and PPAR-γ are also shown for Lut **(** Supplementary Figure S2), RA (Supplementary Figure S3), and RS (Supplementary Figure S4). The grid box coordinates for the specific proteins are presented in Supplementary Table S2 of the Supplementary Material.

### 3.10 *In vitro* enzymatic measurement of α-amylase activity

**FIGURE 9:**
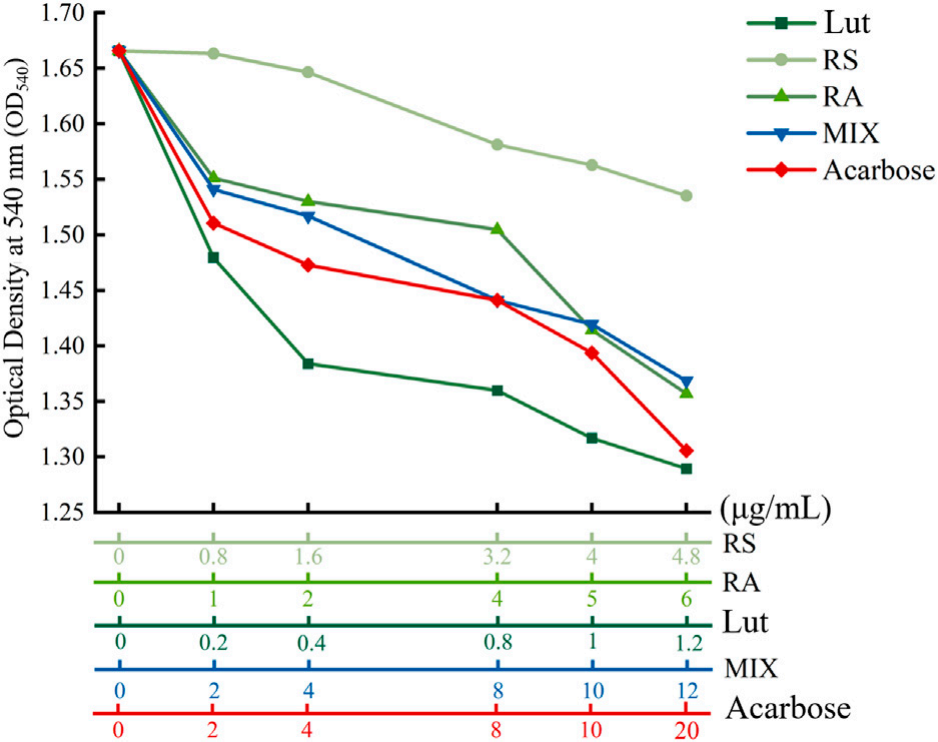
Inhibitory profiles of rosmarinic acid (RA), luteolin (Lut), resveratrol (RS), RLR mixture (MIX), and acarbose on α-amylase activity.

To corroborate the *in vivo* observations, RA, RS, Lut, acarbose, and RLR were tested as the metabolites to measure the alteration of α-amylase activity for understanding the suppressive effects of the RLR mixture on α-amylase activity. The inhibitory patterns of α-amylase activity are shown in Figure 9. Compared to the untreated control, the inhibition of α-amylase activities by RA, RS, Lut, acarbose, and RLR revealed different patterns depending on the concentration used. Furthermore, compared with the reference drug acarbose (a widely prescribed α-glucosidase and α-amylase inhibitor in the clinic), the inhibitory concentration of α-amylase activity for an RLR mixture of 12 µg/mL was equivalent to that of 13.7 µg/mL of acarbose. Additionally, the inhibitory profiles of RA, Lut, and RS on α-amylase activity are associated with the binding ranks of the docking results (kcal/mol) (Supplementary Table S1). This experiment suggests that the RLR mixture possesses an appropriate inhibitory potential for α-amylase activity. It is noted that α-amylase and α-glucosidase are important therapeutic targets for the management of T2DM; the inhibition of these enzymes can result in decrease of postprandial hyperglycemia. This evidence provides an opportunity for a food-based strategy to modulate starch breakdown to glucose, which could contribute to the management of hyperglycemia- and diabetes-related complications.

### 3.11 OSTT

**FIGURE 10:**
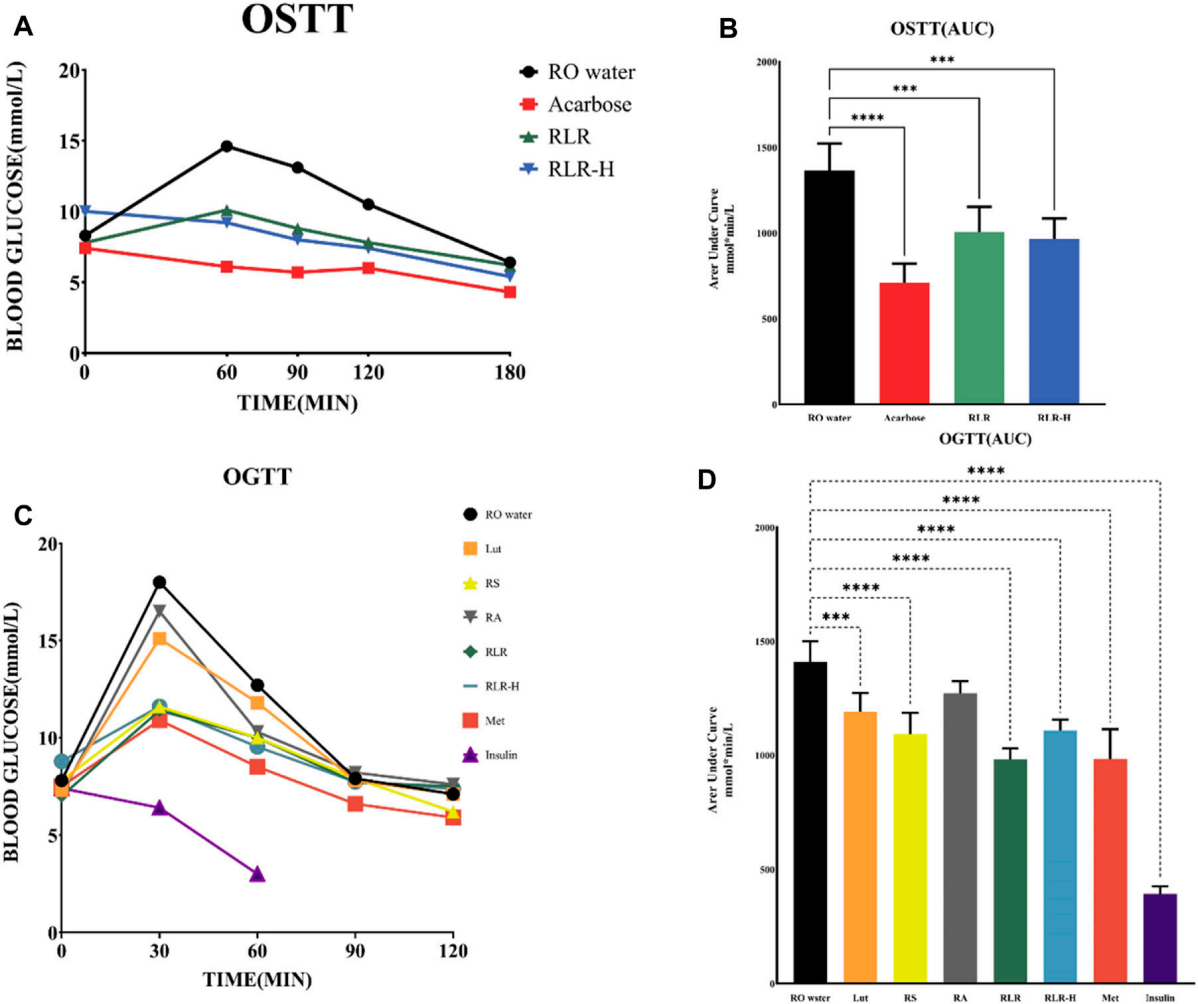
Alteration of blood glucose levels in treatments using rosmarinic acid, luteolin, and resveratrol (RLR) mixture: **(A,B)** oral starch tolerance test (OSTT) and **(C,D)** oral glucose tolerance test (OGTT). Acarbose: 10 mg/kg, Lut: 6.5 mg/kg bodyweight (Bwt), RS: 88 mg/kg Bwt, RA: 50.5 mg/kg Bwt, RLR: 100 mg/kg Bwt, metformin (Met): 125 mg/kg, insulin: 0.2 U/mL. ***p < 0.001; ****p < 0.0001 compared with the control.

To investigate the effect of the RLR mixture for starch digestion, two fasting blood glucose levels (RLR, RLR-H) and acarbose were evaluated via the OSTT to determine the inhibitory efficacy. Acarbose was used as the positive control. Compared to the untreated group, the RLR, RLR-H, and acarbose groups showed good trends for reducing blood glucose levels with time, particularly the acarbose group (Figure 10A, 10B). Interestingly, the suppressive efficacy on blood glucose for fasting blood glucose >7 mmol/L in RLR group was similar to that for fasting blood glucose >10 mmol/L in RLR-H group. Moreover, the results showed that the efficacy of the RLR mixture (100 mg/kg) was equipotent to 48.5% of acarbose (10 mg/kg). The blood glucose levels in the OSTT after administering, the RLR mixture demonstrate maximal responses regardless of the fasting blood glucose levels.

### 3.12 OGTT

The OSTT findings revealed that the RLR mixture significantly inhibited starch digestion. To further understand the hypoglycemic efficacies, RA, Lut, RS, RLR, RLR-H, metformin, and insulin were evaluated via the OGTT. The experimental data indicated that RLR and metformin had similar trends for reducing postprandial blood glucose levels compared to the untreated control. Compared to the reference drug metformin, the impact of RLR was generally equipotent to 78.5% of metformin (125 mg/kg Bwt) (Figures 10C, D). Interestingly, the results illustrated that the RLR mixture lowered blood glucose levels without causing hypoglycemia, implying that this phytomixture would be a good fit for antidiabetes. Here, the abilities of RA, Lut, and RS to bind effectively with the twelve protein targets (α-amylase, α-glucosidase, pancreatic lipase, SGLT-2, AMPK, glucokinase, aldose reductase, acetylcholinesterase, acetylcholine M2 receptor, GLP-1R, DPP-IV, and PPAR-γ) could be pivotal in the treatment of T2DM, in addition to the link capabilities of the metabolites to interact more strongly than the standard drugs with the individual receptors.

## 4 Discussion

This study presents proof-of-concept evaluations for the use of network pharmacology studies and multitarget *in silico* screening to reveal that the RLR mixture has proven hypoglycemic effects. Notably, by reevaluating the blood glucose lowering efficacy of the RLR mixture along with *in vitro* and *in vivo* (OSTT and OGTT) validations for the combined metabolites, this study demonstrates that the mixture has satisfactory performance against diabetes. RA, Lut, and RS are all hydrophobic and water-insoluble natural polyphenolic compounds that frequently show low bioavailability, poor systemic delivery, and low efficacy. Generally, the poor water solubility, rapid decomposition, and short serum half-life (low stability) significantly limit their potential in pharmacological applications, particularly in the treatment of DM. Given these defects, several reports have focused on nanoparticle coatings (Imam et al., 2022), metabolite-based nanodrug delivery (Intagliata et al., 2019; Taghipour et al., 2019; Saleem et al., 2022), and chemical modifications like derivative production to enhance the bioavailability when using natural products. Nonetheless, this process usually takes a long time; therefore, developing combinations with other metabolites in the same solubility range while preventing toxicity can help to meet the treatment goals. It is well known that DM is a complex disease involving the alterations of multiple signaling pathways and that hypoglycemic induction is the preferred treatment for diabetes (Niu et al., 2022). To determine the potential antihyperglycemic mechanisms of RLR, a metabolite–target–pathway network was constructed and five candidate target proteins (PPAR, MGAM, DPP4, VEGFA, and IGF1R) were identified. Despite the widespread pharmacological activities of RA, Lut, and RS, particularly in a mixture, we performed molecular dynamics (MD) simulations on the top-3 complexes identified via network analysis and molecular docking to confirm the lowest binding affinity of metabolite–target interactions. Additionally, the synergistic effects of the RLR mixture are under consideration for exploration in a future study. The ligand–protein interactions and molecular mechanism of the signal pathway via Western blot analysis are also under investigation for understanding the pharmacological value of the mixture in preclinical tests.

### 4.1 Antihyperglycemic effects of RA, Lut, and RS via suppression of digestive enzymes

Given the high prevalence of T2DM, various therapeutic approaches have been attempted for its management, including inhibition of key enzymes such as α-glucosidase and α-amylase. Starch is absorbed by the hydrolytic action of α-amylase, followed by the action of the intestinal α-glucosidase enzyme. Consequently, α-amylase inhibitors that modulate blood glucose levels after meals may be considered effective chemotherapeutic tools to treat diabetes. α-Glucosidase is a carbohydrate-hydrolyzing enzyme secreted by the intestinal chorionic epithelium; the inhibition of this enzyme promotes delayed carbohydrate digestion, thereby preventing excessive glucose absorption (You et al., 2012). Currently, α-glucosidase inhibitors, such as acarbose and nojirimycin, are successfully used to control the glucose levels of diabetic patients. Acarbose has been reported to cause several undesirable side effects, such as abdominal pain, flatulence, and diarrhea (Israili, 2011). The usefulness of many synthetic antidiabetic agents is constrained by their side effects and limited effectiveness. The main advantages of using natural metabolites as alternatives to prescription hypoglycemic drugs include fewer side effects, easy availability, presence of numerous bioactive compounds, and possible multiple protein targets in a single compound that can lower blood glucose levels (Pereira et al., 2019). α-Amylase and α-glucosidase are important therapeutic targets for the management of T2DM; the inhibition of these enzymes through phytochemicals such as RA can reduce postprandial hyperglycemia (Tolmie et al., 2021). RA has been shown to have α-glucosidase inhibitory activity (IC_50_ of 0.23 ± 0.01 mg/mL) (Zhu et al., 2014). The IC_50_ value for Lut is 339.4 ± 16.3 μM against α-glucosidase activity (Zhang et al., 2017). RS having an IC_50_ value of 47.93 ± 5.21 μM has been observed to have a weaker effect on α-amylase than the effect of acarbose (4.60 ± 1.26 μM); however, RS has been found a significant effect on the inhibition of α-glucosidase (32.23 ± 0.56 μM). The IC_50_ value of RS (5.64 ± 0.01 μM) compared to that of Diprotin A (7.21 ± 0.02 μM) indicates that RS may have significantly inhibitory effects on the DPP-IV enzyme (Khalid et al., 2022). The RLR mixture showed good inhibition of α-amylase activity in the present work (Figure 10), revealing the antihyperglycemic potential of this combination.

### 4.2 Antihyperglycemic effects of RA, Lut, and RS

RA is a secondary metabolite and polyphenol that demonstrates antioxidant, anti-inflammatory, anticancer, immunomodulatory, neuroprotective, and other beneficial effects on insulin sensitization and skin afflictions (Alagawany et al., 2017). Lut can work against α-amylase and α-glucosidase proteins by inhibiting starch and digesting disaccharides into glucose (Christian and Nagar, 2021; Jugran et al., 2021). Many preclinical reports reveal that Lut has excellent antioxidant, anticancer, neuroprotective, cardioprotective, and anti-inflammatory effects, and various clinical trials have been designed to investigate its therapeutic potential in humans (Taheri et al., 2021).

RS is a member of the stilbene family and a well-known polyphenolic metabolite found in grapes, apples, blueberries, mulberries, peanuts, pistachios, plums, and red wine; it has been found to potentially exhibit antitumor, antiangiogenic, antidiabetic, antiaging, glucose metabolism, antiobesity, immunomodulatory, and cardioprotective activities, in addition to being an antioxidant (Zhang L.-X. et al., 2021). Although there are various *in vitro* and *in vivo* studies illustrating the effectiveness of RS in DM, many clinical trials show that RS has latent benefits in DM patients (Öztürk et al., 2017). RS may improve insulin resistance, lower fasting blood glucose and insulin levels, and attenuate oxidative stress in patients with T2DM (Zhang T. et al., 2021). RS also protects the pancreatic β-cells, increases insulin secretion and glucose homeostasis, decreases insulin resistance, and ameliorates metabolic disorders (Szkudelski and Szkudelska, 2015). A systematic review and meta-analysis has demonstrated that RS has a statistically significant dose–response effect on blood glucose, glycated hemoglobin/hemoglobin (HbA1c), and insulin levels; however, there is insufficient scientific evidence to propose a therapeutic dose in human subjects (García-Martínez et al., 2022). The most potent DPP-IV inhibitors have been found to be RS, Lut, apigenin, and flavones that exhibit hypoglycemic activities at nanomolar concentrations (Singla et al., 2019). Our previous works have demonstrated the antihyperglycemic effects of botanical drugs, plant sources, and natural products based on *in silico* docking relevant to antidiabetic target proteins (α-amylase, α-glucosidase, AMPK, PPAR-γ, DPP-IV, and GLP-1R), which have been further validated by *in vitro* assays and diabetic mice tests (Huang et al., 2019b; Chen T.-H. et al., 2021; Chen S.-P. et al., 2021). In this study, RA, Lut, and RS were found to dock with more clinically therapeutic targets (Supplementary Table S1; Figure 8), suggesting that RLR could be a latent candidate for antihyperglycemic nutraceuticals.

### 4.3 Signal pathways in the antihyperglycemic effects of RA, Lut, and RS

Mitochondrial biogenesis dysfunction has been associated with metabolic disorders, such as obesity and T2DM. A decline in the PGC-1α/AMPK/SIRT-1 signaling pathway seems to be the underlying mechanism for reduced mitochondrial biogenesis in diabetes. The proliferator-activated receptor gamma coactivator-1α (PGC-1α) protein is regulated by two enzymes, namely, AMPK and silent information regulator 1 (SIRT1), which are important in mitochondrial biogenesis.

The current study shows the effects of RA as a regulatory glucose homeostasis agent to increase muscle glucose uptake and AMPK phosphorylation as the targeted approach for combating insulin resistance (Vlavcheski et al., 2017). RA can reduce hyperglycemia and ameliorate insulin sensitivity by decreasing PEPCK expression and increasing GLUT4 expression for glucose uptake (Runtuwene et al., 2016). The levels of blood glucose, HbA1c, advanced glycation end products (AGE), TNF-α, IL-1β, IL-6, NO, p-JNK, P38MAPK, and NF-κB are significantly reduced with concomitant elevation in the plasma insulin level for oral administration of RA (100 mg/kg Bwt) in diabetic rats (Govindaraj and Sorimuthu Pillai, 2015). Supplementation with RA increases the expressions of the mitochondrial biogenesis genes like PGC-1α, SIRT1, and TFAM through activation of AMPK in the skeletal muscles of rats with insulin resistance as well as in L6 myotubes. Furthermore, RA treatment increases glucose uptake and decreases the phosphorylation of serine IRS-1 while increasing the translocation of GLUT 4 (Jayanthy et al., 2017).

Lut prevents the expression of the transcription factor FOXO1 (Christian and Nagar, 2021); when the FOXO1 transcript is blocked, gluconeogenesis is inhibited through the PI3K-AKT pathway (Christian and Nagar, 2021). Recently, Lut has been shown to inhibit DPP-IV, thus prolonging insulin activity by increasing GLP-1 (Singla et al., 2019). In addition, Lut improves glucose intolerance and reduces the expression of gluconeogenesis-associated enzymes in a liver X receptor (LXR) α-dependent manner (Park et al., 2020). The protective effects of RS include regulation of multiple signaling pathways, such as inhibition of oxidative stress and inflammation, enhancement of insulin sensitivity, induction of autophagy, regulation of lipid metabolism, promotion of GLUT4 expression and translocation, and activation of the SIRT1/AMPK signaling axis (Su et al., 2022). RS treatment significantly decreases the levels of proinflammatory cytokines, HbA1c, IL-6, TNF-α, and IL-1β in diabetes in the elderly (Ma and Zhang, 2022). Previous research has shown that RS could relieve insulin resistance through the Sirt1-p-AMPK-p-AKT and Sirt1-p-IRS-1-p-AKT pathways in adipocytes (Chen et al., 2018), thereby promoting glucose uptake through increased membrane accumulation of Glut4.

For the RLR mixture (100 mg/kg Bwt; RA: Lut: RS = 5:1:4), *in vivo* experiments (Figures 10A, B) have shown the potential to relieve insulin resistance by suppressing starch digestion, stimulating metabolism (glucose uptake, absorption, and utilization), and activating insulin sensitivity. Based on literature, it is clear that dietary metabolites can impede diabetic diseases by 1) blocking oxidative stress-inhibiting inflammatory mediators by suppressing Keap1 or activating Nrf2 expression and their downstream targets in the nucleus, including HO-1, SOD, and CAT; 2) mediating Nrf2 signaling through various kinases like GSK3β, PI3/AKT, and MAPK; and 3) modifying epigenetic modulations, such as methylation, at the Nrf2 promoter region. However, further investigations into other upstream signaling molecules like Nrf2 and the effects of metabolites are needed (Thiruvengadam et al., 2021).

According to the data from the PPI analysis (Figure 5), mitogen-activated protein kinase (MAPK) 3 was the hub target of RLR in T2DM treatment. MAPK3 is a common target for T2DM (Maradesha et al., 2023), and evidence has shown that overactivation of MAPK3 can lead to insulin sensitivity impairment, while MAPK3 knockout can undo insulin resistance (Jiao et al., 2013). GO enrichment analysis predicted that RLR alleviated T2DM through multiple BPs, CCs, and MFs, such as responses to oxidative stress, membrane rafts, and signaling receptor activator activities (Figure 6A). KEGG enrichment analysis predicted that RLR could play a therapeutic role in T2DM by regulating the PI3K-AKT signaling pathway (Figures 6B, 7), which plays key roles in essential cellular processes, such as glucose homeostasis and lipid metabolism (Abeyrathna and Su, 2015). Phosphatidylinositol 3'-kinase (PI3K) is initially activated by several signal molecules, such as growth factors and cytokines. The activated PI3K catalyzes phosphatidylinositol 4,5-biphosphate (PIP2) to phosphatidylinositol 3,4,5-triphosphate (PIP3) and subsequently activates RAC serine/threonine–protein kinase (AKT) for further signal cascade (Franke et al., 1997). Under physiological conditions, insulin mediates glucose uptake and reduces gluconeogenesis through the PI3K-AKT signaling pathway. Nevertheless, in energy excessive conditions, the PI3K-AKT signaling pathway is impaired, causing insulin resistance (Huang et al., 2018). The aforementioned network pharmacology study indicates that RLR has great therapeutic potential for T2DM by regulating the PI3K-AKT signaling pathway, especially MAPK3.

## 5 Conclusion

**FIGURE 11:**
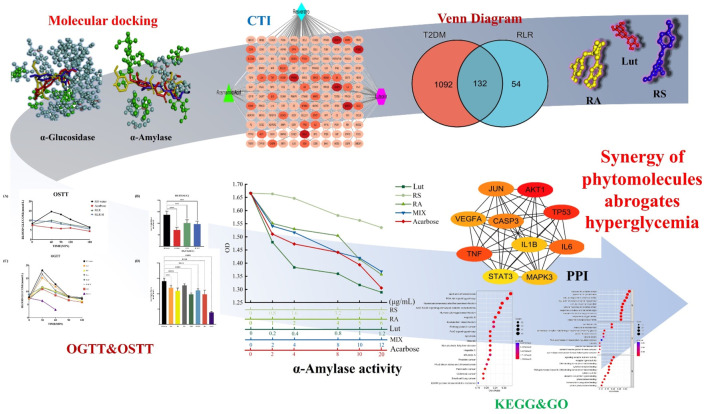
Schematic illustration depicting the synergy of rosmarinic acid (RA), luteolin (Lut), and resveratrol (RS) in the abrogation of hyperglycemia.

Historically, folk and traditional herbal medicines have been popularly used all over the world regardless of the region. Recently, evidence-based, personalized, and precision medicines have become crucial for remedying human diseases through the ethnomedicinal functions of numerous botanical drugs or plant species on diseases (e.g., cancer, diabetes, metabolic syndrome, and microbial infections), free-radical scavengers, and antioxidative stress. In this study, network pharmacology studies, multitarget *in silico* screening, as well as *in vitro* and *in vivo* validations of a combination of metabolites are used to counteract diabetes instead of exploiting synthetic drugs (Figure 11). Multiple predictions obtained via network and docking analyses accompanied by investigation of the underlying mechanisms indicate the potential of the proposed RLR mixture in diabetes treatment. A comprehensive analysis of the efficacy of the RLR mixture on diabetes demonstrated that the mixture holds promising clues regarding the hypoglycemic effect. Furthermore, this study is expected to direct future clinical trials, with important implications for human health.

## Data Availability

The raw data supporting the conclusions of this article will be made available by the authors, without undue reservation.

## Abbreviations

DIO, diet-induced obese; GT, glucose tolerance; IGT, insulin intolerance test; Lut, luteolin; MAPK, mitogen-activated protein kinase; OGTT, oral glucose tolerance test; OSTT, oral starch tolerance test; RA, rosmarinic acid; RS, resveratrol.
