# Supplementary material for: Combination of plant metabolites hinders starch digestion and glucose absorption while facilitating insulin sensitivity to diabetes
Source: Front Pharmacol. 2024 Jun 5;15:1362150. doi: 10.3389/fphar.2024.1362150 (PMC11188438; doi:10.3389/fphar.2024.1362150)
Supplement: Supplementary file 1 [file DataSheet1.zip › Supplementary Material/All supplementary files.docx]

**Combination of Phytocompounds Exerts Compatibly Hindering Starch Digestion and Glucose Absorption with Facilitating Insulin Sensitivity Towards Diabetes**

**Subtitle:** **Synergy of phytomolecules abrogates hyperglycemia**

Xin Huang^a#^, Kaihuang Lin^a#^, Sinian Liu^a^, Junxiong Yang^a^, Haowei Zhao^a^, Xiao-hui Zheng^a^, May-Jywan Tsai^b^, Chun-Sheng Chang^c^, Liyue Huang^a^, Ching-Feng Weng^a,d*^

^a^ Functional Physiology Section, Department of Basic Medical Science, Xiamen Medical College, Xiamen 361023, Fujian, China

^b^ Department of Neurosurgery, Neurological Institute, Taipei Veterans General Hospital, Taipei 11217, Taiwan

^c^ Department of biotechnology and food technology, Southern Taiwan University of Science and Technology, Yungkang City 701, Taiwan.

^d^ Institute of Respiratory Disease, Department of Basic Medical Science, Xiamen Medical College, Xiamen 361023, Fujian, China

# These two authors equally contribute this work

***** Correspondence: Ching-Feng Weng; Institute of Respiratory Disease, Department of Basic Medical Science, Xiamen Medical College, Xiamen361023, Fujian, China. Orcid: 0000-0002-9747-8697; <Tel:+86-0592-6270028>;

Email: 201900051177 @xmmc.edu.cn; [cfweng-cfweng@hotmail.com](mailto:cfweng-cfweng@hotmail.com).

**Table S1.** Rosmarinic acid (RA), luteolin (Lut), and resveratrol (RS) were binding with (A) α-Amylase, α-Glucosidase, and Pancreatic lipase underlined the control of starch digestion; (B) SGLT-2, AMPK, Glucokinase, Aldose reductase, A[cetylcholinesterase](https://www.sciencedirect.com/topics/medicine-and-dentistry/acetylcholinesterase) and Acetylcholine M2 receptor underlined the mediation of glucose absorption; (C) GLP-1R, DPP-IV, PPAR-γ underlined the regulation of insulin sensitivity.

| Targets/ proteins (PDB) | Ligands/ Metabolites | Binding  affinity (Kcal/mol) | H-bond | H-distance (Å) | | Hydrophobic interaction | |  |
| --- | --- | --- | --- | --- | --- | --- | --- | --- |
| (A) Starch digestion | | | | | | | |  |
| α-Amylase  (5U3A) | RA | -7.9 | ARG195 | | 2.33 | | TYR-62 LEU162 |  |
|  |  |  | GLU233 | | 2.29 2.54 | |  |  |
|  |  |  | HIS201 | | 2.69 | |  |  |
|  | Lut | -8.9 | GLN63 | | 2.36 | | TRP59 TYP62 |  |
|  |  |  | ASP197 | | 2.01 | |  |  |
|  |  |  | GLU233 | | 2.97 | |  |  |
|  | RS | -7.4 | ASP197 | | 1.98 | | TRP59 TYP62 |  |
|  | Acarbose | -7.4 | GLU233 | | 2.82 | | TRP59 TYP62 |  |
|  |  |  | HIS201 | | 2.48 | |  |  |
|  |  |  | TRP58 | | 4.39 3.02 | |  |  |
|  |  |  | THR163 | | 2.59 | |  |  |
| α-Amylase  (4GQR) | RA | -7.8 | THR163 | | 3.27 | | TYR62 LEU162 ALA198 |  |
|  |  |  | ASP197 | | 2.15 | |  |  |
|  |  |  | GLU233 | | 1.98 2.18 | |  |  |
|  | Lut | -8.1 | GLU233 | | 2.99 | | TRP59 TYP62 |  |
|  |  |  | ASP277 | | 1.98 2.07 | |  |  |
|  | RS | -7.5 | HIS101 | | 2.41 | | TRP59 TYP62 |  |
|  | Acarbose | -7.2 | THR163 | | 2.60 | | / |  |
|  |  |  | GLN63 | | 2.09 2.57 | |  |  |
|  |  |  | ASP300 | | 2.21 2.88 | |  |  |
|  |  |  |  |  |  |  |  |  |
| α-Glucosidase  (3TOP) | RA | -8.8 | ASP1526 | | 1.98 2.15 | | TYR1521 |  |
|  |  |  | TRP1369 | | 2.14 | |  |  |
|  |  |  | ARG1510 | | 2.42 | |  |  |
|  |  |  | ASP1420 | | 2.58 | |  |  |
|  | Lut | -9.3 | ASP1157 | | 2.68 | | PRO1159 TYR1251 TRP1355 PHE1559 PHE1560 |  |
|  |  |  |  |  |  |  |  |  |
|  | RS | -8.0 | ASP1526 | | 1.97 | | TYP1251 TRP1355 PHE1560 |  |
|  | Acarbose | -7.4 | ARG1377 | | 2.43 | | / |  |
|  |  |  | TYR1251 | | 2.11 | |  |  |
|  |  |  | GLY1365 | | 3.02 | |  |  |
|  |  |  | THR1586 | | 2.47 | |  |  |
|  |  |  | TRP1369 | | 2.78 | |  |  |
|  |  |  | ASP1281 | | 2.19 | |  |  |
|  | Miglitol | -6.1 | LYS1536 | | 2.54 | | / |  |
|  |  |  | GLU1138 | | 2.70 | |  |  |
|  |  |  | GLU1136 | | 1.84 | |  |  |
|  | Voglibose | -6.5 | THR1137 | | 2.66 | | / |  |
|  |  |  | GLU1136 | | 2.30 | |  |  |
|  |  |  | LEU1524 | | 2.46 | |  |  |
|  |  |  | ASN1527 | | 2.61 | |  |  |
|  |  |  | GLN1533 | | 2.00 | |  |  |
|  | Emiglitate | -7.3 | LYS1460 | | 2.28 | | / |  |
|  |  |  | ASP1157 | | 2.72 | |  |  |
|  | 1-Deoxynojirimycin | -5.9 | THR1137 | | 1.93 | | / |  |
|  |  |  | LYS1536 | | 2.22 | |  |  |
|  |  |  | GLN1533 | | 2.70 | |  |  |
|  |  |  | GLU1138 | | 2.59 | |  |  |
| α-Glucosidase  (3L4Y) | RA | -7.1 | ASP327 | | 1.92 | | TYR-299 PHE-575 |  |
|  |  |  | ASP542 | | 2.11 | |  |  |
|  |  |  | TYR50 | | 2.04 | |  |  |
|  | Lut | -7.3 | ASP327 | | 2.42 | | TRP-406 PHE-575 |  |
|  | RS | -7.3 | ASP549 | | 2.74 | | / |  |
|  |  |  | THR546 | | 2.35 | |  |  |
|  |  |  | ASP542 | | 2.00 | |  |  |
|  | Acarbose | -6.8 | ASP549 | | 2.74 | | / |  |
|  |  |  | THR546 | | 2.35 | |  |  |
|  |  |  | ASP542 | | 2.00 | |  |  |
|  | Miglitol | -5.6 | ASP329 | | 2.77 | | / |  |
|  |  |  | ILE328 | | 2.67 | |  |  |
|  |  |  | MET331 | | 2.40 | |  |  |
|  |  |  | ASP340 | | 1.86 | |  |  |
|  |  |  | ARG334 | | 2.49 2.05 | |  |  |
|  | Voglibose | -5.9 | ASP340 | | 2.57 | | / |  |
|  |  |  | ASP343 | | 2.74 | |  |  |
|  |  |  | MET331 | | 1.96 2.01 | |  |  |
|  |  |  | ARG334 | | 1.87 | |  |  |
|  |  |  | GLU300 | | 2.43 | |  |  |
|  | Emiglitate | -7.0 | ASP203 | | 2.26 | | TYR299 TRP406 |  |
|  |  |  | THR204 | | 2.85 | |  |  |
|  |  |  | ARG526 | | 2.81 | |  |  |
|  | 1-Deoxynojirimycin | -5.7 | MET331 | | 2.05 | | / |  |
|  |  |  | GLU333 | | 2.27 | |  |  |
| Pancreatic lipase  (1LPA) | RA | -8.5 | / | | / | | ILE78 TYR114 ALA178 PRO180 PHE215 |  |
|  | Lut | -9.2 | ARG256 | | 2.00 | | PHE77 TYR144 ALA260 HIS263 LEU264 |  |
|  | RS | -8.5 | ARG256 | | 2.00 | | ILE78 TYR114 PRO180 LEU264 |  |
|  | BNG* | -7.3 | PHE77  ASP79  HIS263  ARG256 | | 2.88  2.61  2.40  2.00  2.38  2.87 | | PRO180 |  |
| (B) Glucose absorption | | | | | | | |  |
| SGLT-2 (7VSI) | RA | -9.9 | ASN75 | | 2.08 2.87 | | HIS80 TYR290 |  |
|  |  |  | HIS80 | | 2.28 5.09 | |  |  |
|  |  |  | GLU99 | | 2.52 | |  |  |
|  |  |  | TYP290 | | 2.00 3.92 | |  |  |
|  |  |  | TYP291 | | 2.03 | |  |  |
|  |  |  | GLN457 | | 2.32 | |  |  |
|  | Lut | -9.6 | SER286 | | 2.61 | | LEU84 VAL95 PHE98 PHE453 |  |
|  | RS | -8.5 | ASN75 | | 1.78 | | HIS80 LEU84 PHE98 TYR290 |  |
|  | Dapagliflozin | -7.7 | ARG336 | | 2.49 | | ALA90 |  |
|  |  |  | VAL343 | | 2.78 | |  |  |
|  | Empagliflozin | -9.5 | ASP454 | | 2.70 | | HIS80 VAL95 PHE98 VAL157 TYR290 |  |
|  |  |  | SER362 | | 3.05 | |  |  |
| AMPK (6C9F) | RA | -8.3 | THR106 | | 2.39 | | ILE48 |  |
|  |  |  | ASP108 | | 2.58 | |  |  |
|  | Lut | -8.9 | LEU20 | | 2.31 | | VAL13 ILE48 VAL113 |  |
|  | RS | -8.0 | / | | / | | / |  |
|  | Acadesine | -5.9 | ASP90 | | 1.96 | | VAL113 |  |
|  |  |  | ARG83 | | 2.73 | |  |  |
|  |  |  | ASP108 | | 2.26 2.77 | |  |  |
|  |  |  | LYS31 | | 2.43 | |  |  |
| Glucokinase (3A0I) | RA | -8.7 | GLN98 | | 2.04 2.53 | | TYR214 LEU451 |  |
|  | Lut | -9.2 | TYR61 | | 2.67 | | VAL62 PRO66 ILE159 ILE211 TYR214 VAL452 VAL455 ALA456 |  |
|  | RS | -8.3 | CYS220 | | 2.69 | | PRO66 ILE211 TYR214 MET235 LEU451 VAL455 |  |
|  |  |  | GLN98 | | 2.04 | |  |  |
|  |  |  | LEU451 | | 1.76 | |  |  |
|  | Dorzagliatin | -7.9 | ARG63 | | 2.80 2.35 | | VAL42 ARG63 PRO66 ILE159 ILE211 TYR214 VAL455 |  |
| Aldose reductase (1IEI) | RA | -9.8 | SER210 | | 2.33 | | TYR209 LEU212 LYS262 CYS298 |  |
|  |  |  | TRP20 | | 2.00 | |  |  |
|  |  |  | THR19 | | 2.29 | |  |  |
|  | Lut | -10.1 | ASP216 | | 2.57 | | TRP20 TYR48 TYR209 LYS262 |  |
|  |  |  | GLN183 | | 2.42 | |  |  |
|  | RS | -9.4 | GLN183 | | 2.33 | | TYR48 TYR209 LYS262 |  |
|  | Fidarestat | -7.7 | GLU71 | | 2.86 | | ALA10 LYS11 |  |
|  |  |  | ARG3 | | 2.57 2.89 | |  |  |
|  |  |  | LEU72 | | 2.78 | |  |  |
|  | Epalrestat | -8.3 | SER210 | | 2.18 | | TYR48 TYR209 ILE260 CYS298 |  |
|  |  |  | SER214 | | 2.11 | |  |  |
|  |  |  | LYS21 | | 2.20 | |  |  |
|  |  |  | LYS262 | | 2.53 1.99 | |  |  |
| Acetylcholinesterase (4BDT) | RA | -10.1 | TYR337 | | 2.70 | | TRP86 TYR337 |  |
|  |  |  | ASP74 | | 3.07 | |  |  |
|  |  |  | THR283 | | 2.54 | |  |  |
|  |  |  | GLY120 | | 1.98 | |  |  |
|  |  |  | TYR133 | | 2.35 | |  |  |
|  | Lut | -9.7 | TYR341 | | 1.75 | | TRP86 TYR337 TRP439 |  |
|  |  |  | ASP74 | | 2.97 | |  |  |
|  | RS | -8.6 | GLY120 | | 2.04 | | TRP86 TYP337 |  |
|  |  |  | TYR341 | | 2.59 | |  |  |
|  | Dihydrotanshinone I | -11.8 | SER125 | | 2.75 | | TRP86 TYR337 TRP439 TYR449 |  |
| Acetylcholine M2 receptor (4MQT) | RA | -8.8 | TYR104 | | 1.99 | | VAL407 TRP422 TYR426 |  |
|  |  |  | ILE178 | | 2.42 | |  |  |
|  |  |  | TYR403 | | 2.22 | |  |  |
|  | Lut | -9.6 | TYR80 | | 2.27 | | TYR177 TRP422 |  |
|  |  |  | ASN419 | | 2.62 | |  |  |
|  |  |  | ASN410 | | 2.41 | |  |  |
|  | RS | -8.1 | / | | / | | TRP422 |  |
|  | Lobeline | -8.9 | / | | / | | / |  |
| (C) Insulin sensitivity | | | | | | | |  |
| GLP-1R (7C2E) | RA | -7.5 | ARG310 | | 2.36 2.84 | | PHE230 LEU309 LEU384 |  |
|  |  |  | THR298 | | 2.37 2.75 | |  |  |
|  | Lut | -8 | ARG310 | | 2.77 2.18 | | PHE230 LEU384 |  |
|  |  |  | LEU384 | | 2.84 | |  |  |
|  |  |  | THR298 | | 1.95 2.71 | |  |  |
|  | RS | -7.3 | LEU32 | | 2.85 | | LEU141 LEU201 PHE381 PHE385 |  |
|  |  |  | TYP205 | | 2.74 | |  |  |
|  | RGT1383 | -9.9 | GLN221 | | 2.48 | | LYS197 LEU201 CYS228 PHE230 ILE309 LEU384 |  |
|  |  |  | ARG380 | | 2.62 | |  |  |
| DPP-Ⅳ  (4N8D) | RA | -7.8 | ASP545 | | 3.04 | | TYR547 TRP629 |  |
|  |  |  | VAL546 | | 2.98 | |  |  |
|  |  |  | TRP629 | | 2.43 | |  |  |
|  |  |  | TYR631 | | 2.31 | |  |  |
|  | Lut | -8.4 | TYP631 | | 2.54 | | PHE357 |  |
|  |  |  | ASN710 | | 2.60 | |  |  |
|  | RS | -7.4 | TRP629 | | 2.27 | | TYR547 |  |
|  |  |  | VAL546 | | 2.14 | |  |  |
|  | Sitagliptin | -8.9 | VAL546 | | 2.79 | | TYR547 TRP627 TYR662 TYR666 |  |
|  |  |  | LYS554 | | 2.66 | |  |  |
|  |  |  | ARG125 | | 2.65 | |  |  |
|  | Saxagliptin | -7.9 | ALA707 | | 2.32 | | LYS122 TRP124 PHE240 VAL252 |  |
| PPAR-γ (1WM0) | RA | -8.6 | SER289 | | 1.84 | | ILE281 ARG288 LEU330 MET348 |  |
|  |  |  | SER342 | | 2.10 | |  |  |
|  |  |  | GLY284 | | 2.21 | |  |  |
|  | Lut | -8.6 | HIS266 | | 2.38 | | GLY284 ARG288 LEU330 ILE341 |  |
|  |  |  | ILE281 | | 2.22 | |  |  |
|  |  |  | SER289 | | 2.38 | |  |  |
|  | RS | -7.8 | LEU330 | | 2.98 | | ARG288 LEU330 ILE341 |  |
|  | PLB* | -10.6 | SER342 | | 1.87 | | GLY284 CYS285 ARG288 ALA292 ILE326 MET329 LEU330 LEU333 ILE341 |  |
|  | Chiglitazar | -11.2 | ARG288 | | 2.44 | | LEU228 ILE281 ARG288 MET329 LEU330 LEU333 ILE341 MET348 |  |
|  | Pioglitazone | -8.8 | SER289 | | 2.77 | | ILE249 LEU255 PHE264 ILE281 CYS285 ARG288 ILE241 |  |
| PPAR-γ (4CI5) | RA | -7.9 | GLU291 | | 2.70 | | ARG288 ALA292 ILE326 MET329 |  |
|  |  |  | GLU295 | | 2.26 | |  |  |
|  | Lut | -8.4 | GLU343 | | 3.08 | | ARG288 ALA292 ILE326 MET329 LEU333 |  |
|  | RS | -7.1 | GLU291 | | 2.07 | | ALA292 ILE326 MET329 |  |
|  |  |  | SER342 | | 2.96 | |  |  |
|  | Chiglitazar | -11.7 | / | | / | | CYS285 ARG288 ALA292 ILE326 MET329 LEU330 PHE363 HIS449 LEU453 LEU469 |  |
|  | Pioglitazone | -8.8 | GLU295 | | 2.24 | | ARG288 ALA292 ILE326 LEU330 HIS449 LEU453 LEU465 LEU469 TYR473 |  |
|  | Y1N* | -10.1 | SER342 | | 2.31 | | ARG288 ILE326 LEU330 ILE341 LEU453 LEU465 LEU469 |  |

**The key residues of all protein structures that constitute the putative binding pocket are available**, including (A) α-Amylase (PDB: 5U3A; ASP300, GLU233, ASP197); (PDB: 4GQR; ASP300, GLU233, ASP197); α-Glucosidase (3TOP; PHE1560, PHE1559, ASP1526, ASP1157, ASP1420, ASP1279, TRP1369, TRP1355, TRP1418, TYR1251, HIS1584); (PDB: 3L4Y; ASP542, ASP203, ASP443, ASP327, HIS600, ARG526); Pancreatic lipase (PDB: 1LPA; PRO180, SER152, ARG256, TRP252); (B) AMPK (PDB: 6C9F; THR106, ASN111, ASP108, VAL81, ARG83, VAL11, GLY19, LEU18, LYS31, PHE90, LYS29, ASP88, GLY28, ASN48, LYS51, PHE27); Glucokinase (PDB: 3A0I; ARG63, TYR214, TYR215, GLY97, LEU451); Aldose reductase (PDB: 1IEI; TYR309, LEU300, TRP111, CYS298, TYR48, HIS110); Acetylcholinesterase (PDB: 4BDT; GLY122, ASP74, TYR341, TYR337, TRP439, MET443, PRO446, HIS447, GLU202, SER203, TRP86); Acetylcholine M2 receptor (PDB: 4MQT; TYR104, ASN108, ASN404, TYR403, TYR426, ASP103); (C) GLP-1R (PDB: 7C2E; TRP33, GLY361, PRO358, LEU396, SER155); DPP-Ⅳ (PDB: 4N8D; TYR547, GLU206, GLU205); PPAR-γ (PDB: 1wm0; ILE326, MET329, LEU330, LEU333, ILE341, ARG288, SER342, MET348, PHE264, GLU259, LEU255, ARG280, ILE281, HIS266, GLY284, CYS285); (PDB: 4CI5; PHE226, MET329, LEU330, VAL339, LEU341, LEU355, CYS285, PHE382, PHE287, PHE286, TYR327, ALA292). BNG, nonyl beta-D-glucopyranoside; FFR, 2-[[4-[6-[(4-cyano-2-fluoranyl-phenyl)methoxy]pyridin-2-yl]-3,6-dihydro-2~{H}-pyridin-1-yl]methyl]-3-[[(2~{S})-oxetan-2-yl]methyl]imidazo[4,5-b]pyridine-5-carboxylic acid, 2-[[4-[6-[(4-cyano-2-fluoranyl-phenyl)methoxy]pyridin-2-yl]-3,6-dihydro-2~{H}-pyridin-1-yl]methyl]-3-[[(2~{S})-oxetan-2-yl]methyl]imidazo[4,5-b]pyridine-5-carboxylic acid; Y1N, 2-methyl-2-[4-[2-[4-[(E)-phenyldiazenyl]phenoxy]ethyl]phenoxy]propanoic acid.

**Table S2**. The grid box coordinates for specific proteins.

| **Protein** | **PDB**  **(ID)** | | **Center**  **x** | | | | | | **y** | | | | | **z** | | | | | **Size**  **x** | | | **y** | | | | | | **z** | | | | | | | | |
| --- | --- | --- | --- | --- | --- | --- | --- | --- | --- | --- | --- | --- | --- | --- | --- | --- | --- | --- | --- | --- | --- | --- | --- | --- | --- | --- | --- | --- | --- | --- | --- | --- | --- | --- | --- | --- |
| **(A) Starch digestion** | | | | | | | | | | | | | | | | | | | | | | | | | | | | | | | | | | | | |
| **α-Amylase** | 5U3A | 7.93 | | | | 80.39 | | | | | 152.03 | | | | | 24 | | | | | 17.25 | | | | 22.5 | | | | | | |  |  |  |  |  |
| **α-Glucosidase** | 4GQR | 6.96 | | | | 27.639 | | | | | 49.186 | | | | | 36 | | | | | 36 | | | | 36 | | | | | | |  |  |  |  |  |
| **Pancreatic lipase** | 3TOP | -49.387 | | | | 5.258 | | | | | -62.764 | | | | | 32.25 | | | | | 27 | | | | 27 | | | | | | |  |  |  |  |  |
|  | 3L4Y | 2.37 | | | | 2.431 | | | | | 3.951 | | | | | 75.95 | | | | | 75.95 | | | | 75.95 | | | | | | |  |  |  |  |  |
|  | 1LPA | 0.649 | | | | 29.673 | | | | | 45.843 | | | | | 36 | | | | | 36 | | | | 36 | | | | | | |  |  |  |  |  |
| **(B) Glucose absorption** | | | | | | | | | | | | | | | | | | | | | | | | | | | | | | |  |  |  |  |  |  |
| **SGLT-2** | 7VSI | 2.37 | | | | 2.431 | | | | | 3.951 | | | | | 64.19 | | | | | 71.46 | | | | | 73.88 | | | | | | |  |  |  |  |
| **AMPK** | 6C9F | | -15.96 | | | | 33.3 | | | | | -33.09 | | | | | 30 | | | | | 30 | | | | 30 | | | | | | | |  |  |  |
| **Glucokinase** | 3A0I | | | 2.37 | | | | 2.431 | | | | | 3.951 | | | | | 64.19 | | | | | 71.46 | | | | 73.88 | | | | | | | |  |  |
| **Aldose reductase** | 1IEI | | | 2.37 | | | | 2.431 | | | | | 3.951 | | | | | 45.64 | | | | | 52.53 | | | | 53.39 | | | | | | | |  |  |
| **Acetylcholinesterase** | 4BDT | | | -1.44 | | | | -49.33 | | | | | -56.74 | | | | | 75.25 | | | | | 75.25 | | | | 63.01 | | | | | | | |  |  |
| **Acetylcholine M2 receptor** | 4MQT | | | -1.44 | | | | -49.33 | | | | | -56.74 | | | | | 68.85 | | | | | 49.95 | | | | 85.05 | | | | | | | |  |  |
| **(C) Insulin sensitivity** | | | | | | | | | | | | | | | | | | | | | | | | | | | | | |  |  |  |  |  |  |  |
| **GLP-1R** | 7C2E | | | 39.45 | | | | 44.14 | | | | | 43.82 | | | | | 52.89 | | | | | 998 | | | | 74.67 | | | | | | | |  |  |
| **DPP-IV** | 4N8D | | | 2.37 | | | | 2.431 | | | | | 3.951 | | | | | 98 | | | | | 110 | | | | 118 | | | | | | | |  |  |
| **PPAR-γ** | 1WM0 | | | | 30.04 | | | | | 32.27 | | | | | 13.64 | | | | | 30 | | | | 30 | | | | | 30 | | | | | | |  |
|  | 4CI5 | | | | 14.87 | | | | | 0.73 | | | | | 13.64 | | | | | 40 | | | | 40 | | | | | 40 | | | | | | | |


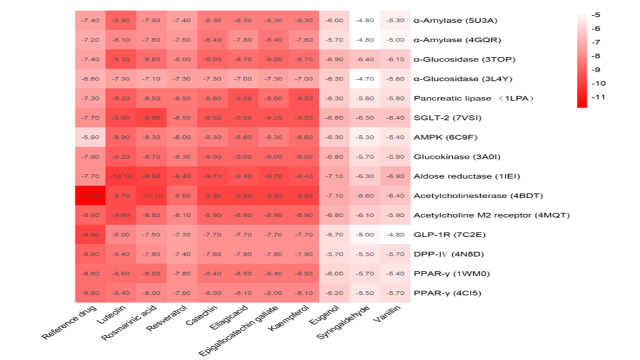


**Fig. S1.** The binding affinity of diverse metabolites docked with various molecular targets via Autodock.

**Fig. S2.** The 2D and 3D depict (A) Underlined the control of starch digestion, Lut bound to the proteins including α-Amylase, α-Glucosidase and Pancreatic lipase. (B) Underlined the mediation of glucose absorption, Lut bound to the proteins including SGLT-2, AMPK, Glucokinase, Aldose reductase, A[cetylcholinesterase](https://www.sciencedirect.com/topics/medicine-and-dentistry/acetylcholinesterase) and Acetylcholine M2 receptor. (C) Underlined the regulation of insulin sensitivity, Lut bound to the proteins including GLP-1R, DPP-IV, and PPAR

**Fig. S3.** The 2D and 3D depict (A) Underlined the control of starch digestion, RA bound to the proteins included α-Amylase, α-Glucosidase and Pancreatic lipase. (B) Underlined the mediation of glucose absorption, RA bound to the proteins included SGLT-2, AMPK, Glucokinase, Aldose reductase, A[cetylcholinesterase](https://www.sciencedirect.com/topics/medicine-and-dentistry/acetylcholinesterase) and Acetylcholine M2 receptor. (C) Underlined the regulation of insulin sensitivity, RA bound to the proteins included GLP-1R, DPP-IV, and PPAR-γ.


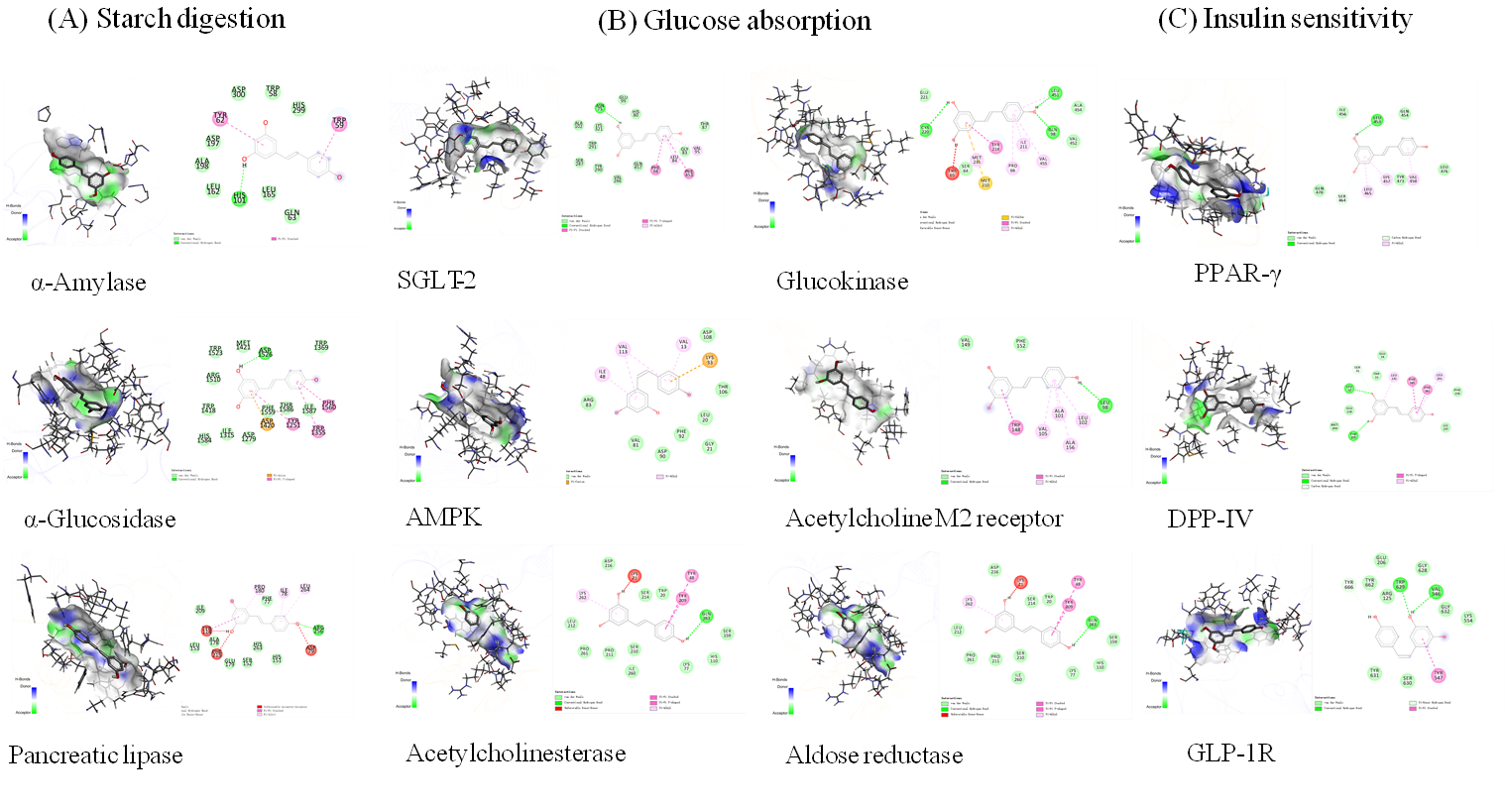
**Fig. S4.** The 2D and 3D depict (A) Underlined the control of starch digestion, RS bound to the proteins included α-Amylase, α-Glucosidase and Pancreatic lipase. (B) Underlined the mediation of glucose absorption, RS bound to the proteins included SGLT-2, AMPK, Glucokinase, Aldose reductase, A[cetylcholinesterase](https://www.sciencedirect.com/topics/medicine-and-dentistry/acetylcholinesterase) and Acetylcholine M2 receptor. (C) Underlined the regulation of insulin sensitivity, RS bound to the proteins included GLP-1R, DPP-IV, and PPAR-
