# Supplementary material for: Combination of plant metabolites hinders starch digestion and glucose absorption while facilitating insulin sensitivity to diabetes
Source: Front Pharmacol. 2024 Jun 5;15:1362150. doi: 10.3389/fphar.2024.1362150 (PMC11188438; doi:10.3389/fphar.2024.1362150)
Supplement: Supplementary file 1 [file DataSheet1.zip › Supplementary Material/supplement_FIG.S1.docx]

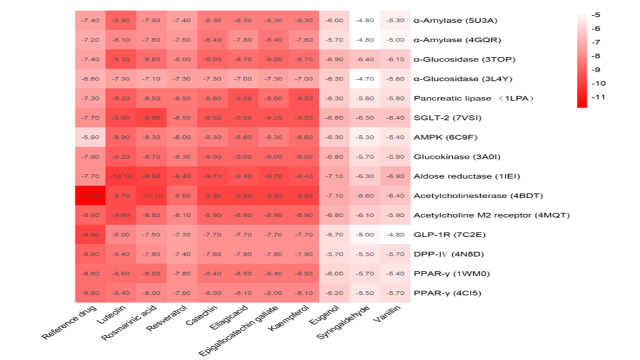


**Fig. S1.** The binding affinity of diverse metabolites docked with various molecular targets via Autodock.
