# Supplementary material for: Combination of plant metabolites hinders starch digestion and glucose absorption while facilitating insulin sensitivity to diabetes
Source: Front Pharmacol. 2024 Jun 5;15:1362150. doi: 10.3389/fphar.2024.1362150 (PMC11188438; doi:10.3389/fphar.2024.1362150)
Supplement: Supplementary file 1 [file DataSheet1.zip › Supplementary Material/supplement_FIG.S3.docx]

**Fig. S3.** The 2D and 3D depict (A) Underlined the control of starch digestion, Lut bound to the proteins including α-Amylase, α-Glucosidase and Pancreatic lipase. (B) Underlined the mediation of glucose absorption, Lut bound to the proteins including SGLT-2, AMPK, Glucokinase, Aldose reductase, A[cetylcholinesterase](https://www.sciencedirect.com/topics/medicine-and-dentistry/acetylcholinesterase) and Acetylcholine M2 receptor. (C) Underlined the regulation of insulin sensitivity, Lut bound to the proteins including GLP-1R, DPP-IV, and PPAR
