# Supplementary material for: Combination of plant metabolites hinders starch digestion and glucose absorption while facilitating insulin sensitivity to diabetes
Source: Front Pharmacol. 2024 Jun 5;15:1362150. doi: 10.3389/fphar.2024.1362150 (PMC11188438; doi:10.3389/fphar.2024.1362150)
Supplement: Supplementary file 1 [file DataSheet1.zip › Supplementary Material/Supplementary Table S1.docx]

**Table S1.** Rosmarinic acid (RA), luteolin (Lut), and resveratrol (RS) were binding with (A) α-Amylase, α-Glucosidase, and Pancreatic lipase underlined the control of starch digestion; (B) SGLT-2, AMPK, Glucokinase, Aldose reductase, A[cetylcholinesterase](https://www.sciencedirect.com/topics/medicine-and-dentistry/acetylcholinesterase) and Acetylcholine M2 receptor underlined the mediation of glucose absorption; (C) GLP-1R, DPP-IV, PPAR-γ underlined the regulation of insulin sensitivity.

| Targets/ proteins (PDB) | Ligands/ Metabolites | Binding  affinity (Kcal/mol) | H-bond | H-distance (Å) | Hydrophobic interaction |  |
| --- | --- | --- | --- | --- | --- | --- |
| (A) Starch digestion | | | | | |  |
| α-Amylase  (5U3A) | RA | -7.9 | ARG195 | 2.33 | TYR-62 LEU162 |  |
|  |  |  | GLU233 | 2.29 2.54 |  |  |
|  |  |  | HIS201 | 2.69 |  |  |
|  | Lut | -8.9 | GLN63 | 2.36 | TRP59 TYP62 |  |
|  |  |  | ASP197 | 2.01 |  |  |
|  |  |  | GLU233 | 2.97 |  |  |
|  | RS | -7.4 | ASP197 | 1.98 | TRP59 TYP62 |  |
|  | Acarbose | -7.4 | GLU233 | 2.82 | TRP59 TYP62 |  |
|  |  |  | HIS201 | 2.48 |  |  |
|  |  |  | TRP58 | 4.39 3.02 |  |  |
|  |  |  | THR163 | 2.59 |  |  |
| α-Amylase  (4GQR) | RA | -7.8 | THR163 | 3.27 | TYR62 LEU162 ALA198 |  |
|  |  |  | ASP197 | 2.15 |  |  |
|  |  |  | GLU233 | 1.98 2.18 |  |  |
|  | Lut | -8.1 | GLU233 | 2.99 | TRP59 TYP62 |  |
|  |  |  | ASP277 | 1.98 2.07 |  |  |
|  | RS | -7.5 | HIS101 | 2.41 | TRP59 TYP62 |  |
|  | Acarbose | -7.2 | THR163 | 2.60 | / |  |
|  |  |  | GLN63 | 2.09 2.57 |  |  |
|  |  |  | ASP300 | 2.21 2.88 |  |  |
|  |  |  |  |  |  |  |
| α-Glucosidase  (3TOP) | RA | -8.8 | ASP1526 | 1.98 2.15 | TYR1521 |  |
|  |  |  | TRP1369 | 2.14 |  |  |
|  |  |  | ARG1510 | 2.42 |  |  |
|  |  |  | ASP1420 | 2.58 |  |  |
|  | Lut | -9.3 | ASP1157 | 2.68 | PRO1159 TYR1251 TRP1355 PHE1559 PHE1560 |  |
|  |  |  |  |  |  |  |
|  | RS | -8.0 | ASP1526 | 1.97 | TYP1251 TRP1355 PHE1560 |  |
|  | Acarbose | -7.4 | ARG1377 | 2.43 | / |  |
|  |  |  | TYR1251 | 2.11 |  |  |
|  |  |  | GLY1365 | 3.02 |  |  |
|  |  |  | THR1586 | 2.47 |  |  |
|  |  |  | TRP1369 | 2.78 |  |  |
|  |  |  | ASP1281 | 2.19 |  |  |
|  | Miglitol | -6.1 | LYS1536 | 2.54 | / |  |
|  |  |  | GLU1138 | 2.70 |  |  |
|  |  |  | GLU1136 | 1.84 |  |  |
|  | Voglibose | -6.5 | THR1137 | 2.66 | / |  |
|  |  |  | GLU1136 | 2.30 |  |  |
|  |  |  | LEU1524 | 2.46 |  |  |
|  |  |  | ASN1527 | 2.61 |  |  |
|  |  |  | GLN1533 | 2.00 |  |  |
|  | Emiglitate | -7.3 | LYS1460 | 2.28 | / |  |
|  |  |  | ASP1157 | 2.72 |  |  |
|  | 1-Deoxynojirimycin | -5.9 | THR1137 | 1.93 | / |  |
|  |  |  | LYS1536 | 2.22 |  |  |
|  |  |  | GLN1533 | 2.70 |  |  |
|  |  |  | GLU1138 | 2.59 |  |  |
| α-Glucosidase  (3L4Y) | RA | -7.1 | ASP327 | 1.92 | TYR-299 PHE-575 |  |
|  |  |  | ASP542 | 2.11 |  |  |
|  |  |  | TYR50 | 2.04 |  |  |
|  | Lut | -7.3 | ASP327 | 2.42 | TRP-406 PHE-575 |  |
|  | RS | -7.3 | ASP549 | 2.74 | / |  |
|  |  |  | THR546 | 2.35 |  |  |
|  |  |  | ASP542 | 2.00 |  |  |
|  | Acarbose | -6.8 | ASP549 | 2.74 | / |  |
|  |  |  | THR546 | 2.35 |  |  |
|  |  |  | ASP542 | 2.00 |  |  |
|  | Miglitol | -5.6 | ASP329 | 2.77 | / |  |
|  |  |  | ILE328 | 2.67 |  |  |
|  |  |  | MET331 | 2.40 |  |  |
|  |  |  | ASP340 | 1.86 |  |  |
|  |  |  | ARG334 | 2.49 2.05 |  |  |
|  | Voglibose | -5.9 | ASP340 | 2.57 | / |  |
|  |  |  | ASP343 | 2.74 |  |  |
|  |  |  | MET331 | 1.96 2.01 |  |  |
|  |  |  | ARG334 | 1.87 |  |  |
|  |  |  | GLU300 | 2.43 |  |  |
|  | Emiglitate | -7.0 | ASP203 | 2.26 | TYR299 TRP406 |  |
|  |  |  | THR204 | 2.85 |  |  |
|  |  |  | ARG526 | 2.81 |  |  |
|  | 1-Deoxynojirimycin | -5.7 | MET331 | 2.05 | / |  |
|  |  |  | GLU333 | 2.27 |  |  |
| Pancreatic lipase  (1LPA) | RA | -8.5 | / | / | ILE78 TYR114 ALA178 PRO180 PHE215 |  |
|  | Lut | -9.2 | ARG256 | 2.00 | PHE77 TYR144 ALA260 HIS263 LEU264 |  |
|  | RS | -8.5 | ARG256 | 2.00 | ILE78 TYR114 PRO180 LEU264 |  |
|  | BNG* | -7.3 | PHE77  ASP79  HIS263  ARG256 | 2.88  2.61  2.40  2.00  2.38  2.87 | PRO180 |  |
| (B) Glucose absorption | | | | | |  |
| SGLT-2 (7VSI) | RA | -9.9 | ASN75 | 2.08 2.87 | HIS80 TYR290 |  |
|  |  |  | HIS80 | 2.28 5.09 |  |  |
|  |  |  | GLU99 | 2.52 |  |  |
|  |  |  | TYP290 | 2.00 3.92 |  |  |
|  |  |  | TYP291 | 2.03 |  |  |
|  |  |  | GLN457 | 2.32 |  |  |
|  | Lut | -9.6 | SER286 | 2.61 | LEU84 VAL95 PHE98 PHE453 |  |
|  | RS | -8.5 | ASN75 | 1.78 | HIS80 LEU84 PHE98 TYR290 |  |
|  | Dapagliflozin | -7.7 | ARG336 | 2.49 | ALA90 |  |
|  |  |  | VAL343 | 2.78 |  |  |
|  | Empagliflozin | -9.5 | ASP454 | 2.70 | HIS80 VAL95 PHE98 VAL157 TYR290 |  |
|  |  |  | SER362 | 3.05 |  |  |
| AMPK (6C9F) | RA | -8.3 | THR106 | 2.39 | ILE48 |  |
|  |  |  | ASP108 | 2.58 |  |  |
|  | Lut | -8.9 | LEU20 | 2.31 | VAL13 ILE48 VAL113 |  |
|  | RS | -8.0 | / | / | / |  |
|  | Acadesine | -5.9 | ASP90 | 1.96 | VAL113 |  |
|  |  |  | ARG83 | 2.73 |  |  |
|  |  |  | ASP108 | 2.26 2.77 |  |  |
|  |  |  | LYS31 | 2.43 |  |  |
| Glucokinase (3A0I) | RA | -8.7 | GLN98 | 2.04 2.53 | TYR214 LEU451 |  |
|  | Lut | -9.2 | TYR61 | 2.67 | VAL62 PRO66 ILE159 ILE211 TYR214 VAL452 VAL455 ALA456 |  |
|  | RS | -8.3 | CYS220 | 2.69 | PRO66 ILE211 TYR214 MET235 LEU451 VAL455 |  |
|  |  |  | GLN98 | 2.04 |  |  |
|  |  |  | LEU451 | 1.76 |  |  |
|  | Dorzagliatin | -7.9 | ARG63 | 2.80 2.35 | VAL42 ARG63 PRO66 ILE159 ILE211 TYR214 VAL455 |  |
| Aldose reductase (1IEI) | RA | -9.8 | SER210 | 2.33 | TYR209 LEU212 LYS262 CYS298 |  |
|  |  |  | TRP20 | 2.00 |  |  |
|  |  |  | THR19 | 2.29 |  |  |
|  | Lut | -10.1 | ASP216 | 2.57 | TRP20 TYR48 TYR209 LYS262 |  |
|  |  |  | GLN183 | 2.42 |  |  |
|  | RS | -9.4 | GLN183 | 2.33 | TYR48 TYR209 LYS262 |  |
|  | Fidarestat | -7.7 | GLU71 | 2.86 | ALA10 LYS11 |  |
|  |  |  | ARG3 | 2.57 2.89 |  |  |
|  |  |  | LEU72 | 2.78 |  |  |
|  | Epalrestat | -8.3 | SER210 | 2.18 | TYR48 TYR209 ILE260 CYS298 |  |
|  |  |  | SER214 | 2.11 |  |  |
|  |  |  | LYS21 | 2.20 |  |  |
|  |  |  | LYS262 | 2.53 1.99 |  |  |
| Acetylcholinesterase (4BDT) | RA | -10.1 | TYR337 | 2.70 | TRP86 TYR337 |  |
|  |  |  | ASP74 | 3.07 |  |  |
|  |  |  | THR283 | 2.54 |  |  |
|  |  |  | GLY120 | 1.98 |  |  |
|  |  |  | TYR133 | 2.35 |  |  |
|  | Lut | -9.7 | TYR341 | 1.75 | TRP86 TYR337 TRP439 |  |
|  |  |  | ASP74 | 2.97 |  |  |
|  | RS | -8.6 | GLY120 | 2.04 | TRP86 TYP337 |  |
|  |  |  | TYR341 | 2.59 |  |  |
|  | Dihydrotanshinone I | -11.8 | SER125 | 2.75 | TRP86 TYR337 TRP439 TYR449 |  |
| Acetylcholine M2 receptor (4MQT) | RA | -8.8 | TYR104 | 1.99 | VAL407 TRP422 TYR426 |  |
|  |  |  | ILE178 | 2.42 |  |  |
|  |  |  | TYR403 | 2.22 |  |  |
|  | Lut | -9.6 | TYR80 | 2.27 | TYR177 TRP422 |  |
|  |  |  | ASN419 | 2.62 |  |  |
|  |  |  | ASN410 | 2.41 |  |  |
|  | RS | -8.1 | / | / | TRP422 |  |
|  | Lobeline | -8.9 | / | / | / |  |
| (C) Insulin sensitivity | | | | | |  |
| GLP-1R (7C2E) | RA | -7.5 | ARG310 | 2.36 2.84 | PHE230 LEU309 LEU384 |  |
|  |  |  | THR298 | 2.37 2.75 |  |  |
|  | Lut | -8 | ARG310 | 2.77 2.18 | PHE230 LEU384 |  |
|  |  |  | LEU384 | 2.84 |  |  |
|  |  |  | THR298 | 1.95 2.71 |  |  |
|  | RS | -7.3 | LEU32 | 2.85 | LEU141 LEU201 PHE381 PHE385 |  |
|  |  |  | TYP205 | 2.74 |  |  |
|  | RGT1383 | -9.9 | GLN221 | 2.48 | LYS197 LEU201 CYS228 PHE230 ILE309 LEU384 |  |
|  |  |  | ARG380 | 2.62 |  |  |
| DPP-Ⅳ  (4N8D) | RA | -7.8 | ASP545 | 3.04 | TYR547 TRP629 |  |
|  |  |  | VAL546 | 2.98 |  |  |
|  |  |  | TRP629 | 2.43 |  |  |
|  |  |  | TYR631 | 2.31 |  |  |
|  | Lut | -8.4 | TYP631 | 2.54 | PHE357 |  |
|  |  |  | ASN710 | 2.60 |  |  |
|  | RS | -7.4 | TRP629 | 2.27 | TYR547 |  |
|  |  |  | VAL546 | 2.14 |  |  |
|  | Sitagliptin | -8.9 | VAL546 | 2.79 | TYR547 TRP627 TYR662 TYR666 |  |
|  |  |  | LYS554 | 2.66 |  |  |
|  |  |  | ARG125 | 2.65 |  |  |
|  | Saxagliptin | -7.9 | ALA707 | 2.32 | LYS122 TRP124 PHE240 VAL252 |  |
| PPAR-γ (1WM0) | RA | -8.6 | SER289 | 1.84 | ILE281 ARG288 LEU330 MET348 |  |
|  |  |  | SER342 | 2.10 |  |  |
|  |  |  | GLY284 | 2.21 |  |  |
|  | Lut | -8.6 | HIS266 | 2.38 | GLY284 ARG288 LEU330 ILE341 |  |
|  |  |  | ILE281 | 2.22 |  |  |
|  |  |  | SER289 | 2.38 |  |  |
|  | RS | -7.8 | LEU330 | 2.98 | ARG288 LEU330 ILE341 |  |
|  | PLB* | -10.6 | SER342 | 1.87 | GLY284 CYS285 ARG288 ALA292 ILE326 MET329 LEU330 LEU333 ILE341 |  |
|  | Chiglitazar | -11.2 | ARG288 | 2.44 | LEU228 ILE281 ARG288 MET329 LEU330 LEU333 ILE341 MET348 |  |
|  | Pioglitazone | -8.8 | SER289 | 2.77 | ILE249 LEU255 PHE264 ILE281 CYS285 ARG288 ILE241 |  |
| PPAR-γ (4CI5) | RA | -7.9 | GLU291 | 2.70 | ARG288 ALA292 ILE326 MET329 |  |
|  |  |  | GLU295 | 2.26 |  |  |
|  | Lut | -8.4 | GLU343 | 3.08 | ARG288 ALA292 ILE326 MET329 LEU333 |  |
|  | RS | -7.1 | GLU291 | 2.07 | ALA292 ILE326 MET329 |  |
|  |  |  | SER342 | 2.96 |  |  |
|  | Chiglitazar | -11.7 | / | / | CYS285 ARG288 ALA292 ILE326 MET329 LEU330 PHE363 HIS449 LEU453 LEU469 |  |
|  | Pioglitazone | -8.8 | GLU295 | 2.24 | ARG288 ALA292 ILE326 LEU330 HIS449 LEU453 LEU465 LEU469 TYR473 |  |
|  | Y1N* | -10.1 | SER342 | 2.31 | ARG288 ILE326 LEU330 ILE341 LEU453 LEU465 LEU469 |  |

**The key residues of all protein structures that constitute the putative binding pocket are available**, including (A) α-Amylase (PDB: 5U3A; ASP300, GLU233, ASP197); (PDB: 4GQR; ASP300, GLU233, ASP197); α-Glucosidase (3TOP; PHE1560, PHE1559, ASP1526, ASP1157, ASP1420, ASP1279, TRP1369, TRP1355, TRP1418, TYR1251, HIS1584); (PDB: 3L4Y; ASP542, ASP203, ASP443, ASP327, HIS600, ARG526); Pancreatic lipase (PDB: 1LPA; PRO180, SER152, ARG256, TRP252); (B) AMPK (PDB: 6C9F; THR106, ASN111, ASP108, VAL81, ARG83, VAL11, GLY19, LEU18, LYS31, PHE90, LYS29, ASP88, GLY28, ASN48, LYS51, PHE27); Glucokinase (PDB: 3A0I; ARG63, TYR214, TYR215, GLY97, LEU451); Aldose reductase (PDB: 1IEI; TYR309, LEU300, TRP111, CYS298, TYR48, HIS110); Acetylcholinesterase (PDB: 4BDT; GLY122, ASP74, TYR341, TYR337, TRP439, MET443, PRO446, HIS447, GLU202, SER203, TRP86); Acetylcholine M2 receptor (PDB: 4MQT; TYR104, ASN108, ASN404, TYR403, TYR426, ASP103); (C) GLP-1R (PDB: 7C2E; TRP33, GLY361, PRO358, LEU396, SER155); DPP-Ⅳ (PDB: 4N8D; TYR547, GLU206, GLU205); PPAR-γ (PDB: 1wm0; ILE326, MET329, LEU330, LEU333, ILE341, ARG288, SER342, MET348, PHE264, GLU259, LEU255, ARG280, ILE281, HIS266, GLY284, CYS285); (PDB: 4CI5; PHE226, MET329, LEU330, VAL339, LEU341, LEU355, CYS285, PHE382, PHE287, PHE286, TYR327, ALA292). BNG, nonyl beta-D-glucopyranoside; FFR, 2-[[4-[6-[(4-cyano-2-fluoranyl-phenyl)methoxy]pyridin-2-yl]-3,6-dihydro-2~{H}-pyridin-1-yl]methyl]-3-[[(2~{S})-oxetan-2-yl]methyl]imidazo[4,5-b]pyridine-5-carboxylic acid, 2-[[4-[6-[(4-cyano-2-fluoranyl-phenyl)methoxy]pyridin-2-yl]-3,6-dihydro-2~{H}-pyridin-1-yl]methyl]-3-[[(2~{S})-oxetan-2-yl]methyl]imidazo[4,5-b]pyridine-5-carboxylic acid; Y1N, 2-methyl-2-[4-[2-[4-[(E)-phenyldiazenyl]phenoxy]ethyl]phenoxy]propanoic acid.
