# Supplementary material for: Combination of plant metabolites hinders starch digestion and glucose absorption while facilitating insulin sensitivity to diabetes
Source: Front Pharmacol. 2024 Jun 5;15:1362150. doi: 10.3389/fphar.2024.1362150 (PMC11188438; doi:10.3389/fphar.2024.1362150)
Supplement: Supplementary file 1 [file DataSheet1.zip › Supplementary Material/Supplementary Table S2.docx]

**Table S2**. The grid box coordinates for specific proteins

| **Protein** | **PDB**  **(ID)** | | **Center**  **x** | | | | | | **y** | | | | | **z** | | | | | **Size**  **x** | | | | | **y** | | | | | **z** | | | | | | | | |
| --- | --- | --- | --- | --- | --- | --- | --- | --- | --- | --- | --- | --- | --- | --- | --- | --- | --- | --- | --- | --- | --- | --- | --- | --- | --- | --- | --- | --- | --- | --- | --- | --- | --- | --- | --- | --- | --- |
| **(A) Starch digestion** | | | | | | | | | | | | | | | | | | | | | | | | | | | | | | | | | | | | | |
| **α-Amylase** | 5U3A | 7.93 | | | | 80.39 | | | | | 152.03 | | | | | 24 | | | | | 17.25 | | | | | 22.5 | | | | | | |  |  |  |  |  |
| **α-Glucosidase** | 4GQR | 6.96 | | | | 27.639 | | | | | 49.186 | | | | | 36 | | | | | 36 | | | | | 36 | | | | | | |  |  |  |  |  |
| **Pancreatic lipase** | 3TOP | -49.387 | | | | 5.258 | | | | | -62.764 | | | | | 32.25 | | | | | 27 | | | | | 27 | | | | | | |  |  |  |  |  |
|  | 3L4Y | 2.37 | | | | 2.431 | | | | | 3.951 | | | | | 75.95 | | | | | 75.95 | | | | | 75.95 | | | | | | |  |  |  |  |  |
|  | 1LPA | 0.649 | | | | 29.673 | | | | | 45.843 | | | | | 36 | | | | | 36 | | | | | 36 | | | | | | |  |  |  |  |  |
| **(B) Glucose absorption** | | | | | | | | | | | | | | | | | | | | | | | | | | | | | | | |  |  |  |  |  |  |
| **SGLT-2** | 7VSI | 2.37 | | | | 2.431 | | | | | 3.951 | | | | | 64.19 | | | | | 71.46 | | | | | | 73.88 | | | | | | |  |  |  |  |
| **AMPK** | 6C9F | | -15.96 | | | | 33.3 | | | | | -33.09 | | | | | 30 | | | | | 30 | | | | | 30 | | | | | | | |  |  |  |
| **Glucokinase** | 3A0I | | | 2.37 | | | | 2.431 | | | | | 3.951 | | | | | 64.19 | | | | | 71.46 | | | | | 73.88 | | | | | | | |  |  |
| **Aldose reductase** | 1IEI | | | 2.37 | | | | 2.431 | | | | | 3.951 | | | | | 45.64 | | | | | 52.53 | | | | | 53.39 | | | | | | | |  |  |
| **Acetylcholinesterase** | 4BDT | | | -1.44 | | | | -49.33 | | | | | -56.74 | | | | | 75.25 | | | | | 75.25 | | | | | 63.01 | | | | | | | |  |  |
| **Acetylcholine M2 receptor** | 4MQT | | | -1.44 | | | | -49.33 | | | | | -56.74 | | | | | 68.85 | | | | | 49.95 | | | | | 85.05 | | | | | | | |  |  |
| **(C) Insulin sensitivity** | | | | | | | | | | | | | | | | | | | | | | | | | | | | | | |  |  |  |  |  |  |  |
| **GLP-1R** | 7C2E | | | 39.45 | | | | 44.14 | | | | | 43.82 | | | | | 52.89 | | | | | 998 | | | | | 74.67 | | | | | | | |  |  |
| **DPP-IV** | 4N8D | | | 2.37 | | | | 2.431 | | | | | 3.951 | | | | | 98 | | | | | 110 | | | | | 118 | | | | | | | |  |  |
| **PPAR-γ** | 1WM0 | | | | 30.04 | | | | | 32.27 | | | | | 13.64 | | | | | 30 | | | | | 30 | | | | | 30 | | | | | | |  |
|  | 4CI5 | | | | 14.87 | | | | | 0.73 | | | | | 13.64 | | | | | 40 | | | | | 40 | | | | | 40 | | | | | | | |
